# Two Distinct Thermodynamic Gradients for Cellular Metalation of Vitamin B_12_

**DOI:** 10.1021/jacsau.3c00119

**Published:** 2023-05-10

**Authors:** Tessa R. Young, Evelyne Deery, Andrew W. Foster, Maria Alessandra Martini, Deenah Osman, Martin J. Warren, Nigel J. Robinson

**Affiliations:** † Department of Biosciences, Durham University, Durham DH1 3LE, U.K.; ‡ Department of Chemistry, Durham University, Durham DH1 3LE, U.K.; § School of Biosciences, University of Kent, Canterbury CT2 7NJ, U.K.; ∥ Department of Inorganic Spectroscopy, Max Planck Institute for Chemical Energy Conversion, 45470 Mülheim an der Ruhr, Germany; ⊥ Quadram Institute Bioscience, Norwich Research Park, Norwich NR4 7UQ, U.K.

**Keywords:** bioinorganic chemistry, metalation, metalloprotein, chelatase, metallochaperone, GTPase, vitamin B_12_

## Abstract

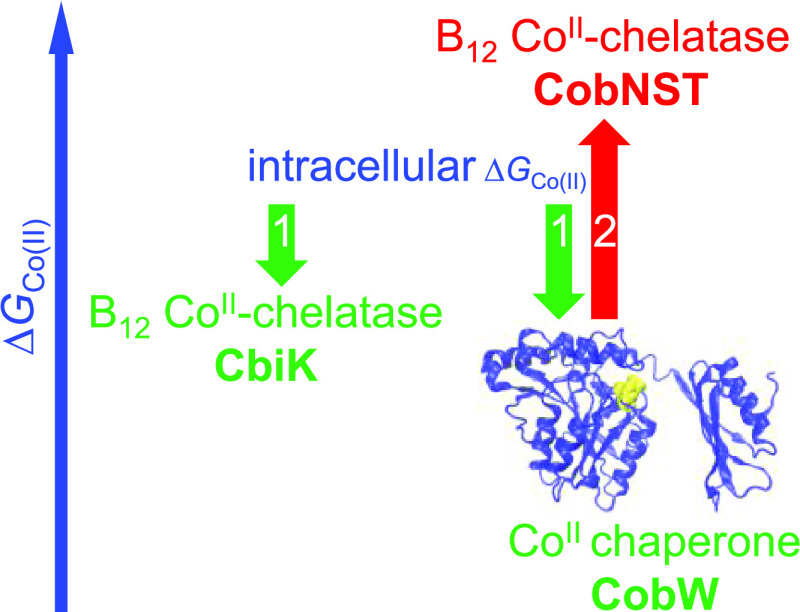

The acquisition of Co^II^ by the corrin component of vitamin B_12_ follows one of two distinct pathways, referred to as early or late Co^II^ insertion. The late insertion pathway exploits a Co^II^ metallochaperone (CobW) from the COG0523 family of G3E GTPases, while the early insertion pathway does not. This provides an opportunity to contrast the thermodynamics of metalation in a metallochaperone-requiring and a metallochaperone-independent pathway. In the metallochaperone-independent route, sirohydrochlorin (SHC) associates with the CbiK chelatase to form Co^II^-SHC. Co^II^-buffered enzymatic assays indicate that SHC binding enhances the thermodynamic gradient for Co^II^ transfer from the cytosol to CbiK. In the metallochaperone-dependent pathway, hydrogenobyrinic acid *a,c*-diamide (HBAD) associates with the CobNST chelatase to form Co^II^-HBAD. Here, Co^II^-buffered enzymatic assays indicate that Co^II^ transfer from the cytosol to HBAD-CobNST must somehow traverse a highly unfavorable thermodynamic gradient for Co^II^ binding. Notably, there is a favorable gradient for Co^II^ transfer from the cytosol to the Mg^II^GTP-CobW metallochaperone, but further transfer of Co^II^ from the GTP-bound metallochaperone to the HBAD-CobNST chelatase complex is thermodynamically unfavorable. However, after nucleotide hydrolysis, Co^II^ transfer from the chaperone to the chelatase complex is calculated to become favorable. These data reveal that the CobW metallochaperone can overcome an unfavorable thermodynamic gradient for Co^II^ transfer from the cytosol to the chelatase by coupling this process to GTP hydrolysis.

## Introduction

Most metalloenzymes (∼70%) are thought to acquire metal ions directly from nonspecific exchangeable sites inside cells with a further 25% containing preassembled metal cofactors.^1^ Cofactors such as heme, cofactor F_430_, chlorophyll, and vitamin B_12_ acquire Fe^II^, Ni^II^, Mg^II^, and Co^II^ from chelatases.^2,3^ Some chelatases such as CbiK for vitamin B_12_ biosynthesis are thought to directly acquire metal (Co^II^) from the exchangeable cytosolic sites, while others such as CobNST from the alternative vitamin B_12_ biosynthesis pathway acquire metal (Co^II^) from specific metallochaperones (in this case, CobW), which, in turn, acquire metal from the exchangeable sites.^4^ How is Co^II^ driven from nonspecific intracellular exchangeable sites to vitamin B_12_ either via a chelatase alone (CbiK) or via a chelatase and a metallochaperone (CobNST and CobW)?^5^

**Figure 1:**
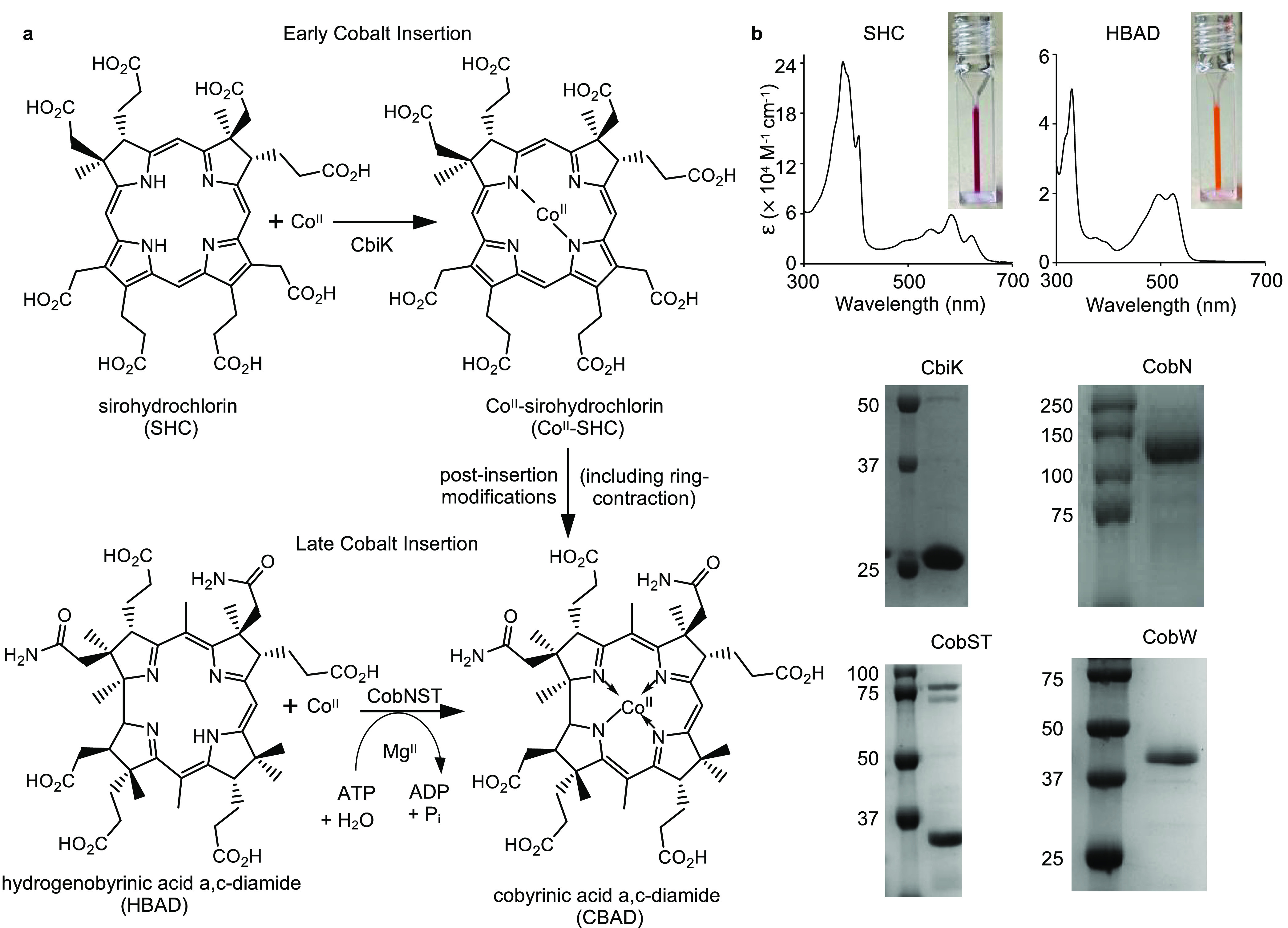
Enzyme-catalyzed cobalt insertion reactions for the biosynthesis of vitamin B_12_. (a) The two biosynthetic pathways for vitamin B_12_ involve early- and late-stage insertion of the catalytic cobalt ion, respectively.^2,7^ Co^II^ insertion into the porphyrin substrate sirohydrochlorin (SHC) to form cobalt-sirohydrochlorin (Co^II^-SHC) is catalyzed by the cobaltochelatase CbiK.^17^ Co^II^ insertion into the ring-contracted substrate hydrogenobyrinic acid *a*,*c*-diamide (HBAD) to form cobyrinic acid *a*,*c*-diamide (CBAD) is catalyzed by the cobaltochelatase CobNST, which requires energy input from ATP hydrolysis and an additional metallochaperone (CobW) for Co^II^ supply *in vivo.*(4,9) (b) Absorbance spectra of isolated substrates and SDS-PAGE analyses of isolated enzymes (full-length gel images shown in Figure S1).

CbiK inserts Co^II^ into the substrate sirohydrochlorin (SHC) at an early stage (before ring contraction) in the vitamin B_12_ synthesis pathway first discovered in anaerobic bacteria (Figure 1a).^6^ CbiK is a relatively small protein (∼29 kDa in *Salmonella enterica* serovar Typhimurium strain 1344, referred to as *Salmonella* hereafter) that works without accessory proteins or nucleotide cofactors.^7^ In the alternative (aerobic) pathway, Co^II^ is inserted at a late stage into the ring-contracted vitamin B_12_ precursor hydrogenobyrinic acid *a*,*c*-diamide (HBAD) by the multicomponent chelatase CobNST (13 subunits), which, in common with the structurally related magnesium chelatase for chlorophyll biosynthesis,^8^ couples metal insertion with the hydrolysis of ATP (Figure 1a).^9,10^ The additional chaperone (CobW) is, for some reason, also used to supply Co^II^ to CobNST *in vivo.*(4)

Vitamin B_12_ is an essential dietary component but absent in plants. Supplements are therefore recommended for vegans. While the complete chemical synthesis of vitamin B_12_ has been achieved, these elaborate natural product syntheses are unsuitable for commercial-scale production.^11,12^ Instead, vitamin B_12_ is manufactured exclusively through bioprocessing. However, the synthesis of vitamin B_12_ is restricted to a subset of bacteria and archaea, which does not include organisms such as *Escherichia coli* that are commonly used in manufacturing. When the biosynthetic pathway involving CobW and CobNST was introduced into *E. coli*, B_12_ production stalled at the point of Co^II^ insertion in standard culture media.^4^ Intriguingly, the F_430_ pathway from methanobacteria introduced into *E. coli* also stalled at the point of metal (Ni^II^) insertion even in Ni^II^-supplemented media, and this was overcome by introducing genes encoding an additional Ni^II^ importer.^13^ These observations suggest that exchangeable metals could be maintained at different availabilities in different organisms leading to mismatches between heterologously introduced proteins and host cells such as *E. coli*. An understanding of the thermodynamic gradients that supply metals to proteins and cofactors in a cellular context is needed to enable optimization and exploitation of metalloproteins in engineering biology.

Intracellular metal availabilities have been estimated as free energies for forming metal complexes, by calibrating the responses of bacterial metal sensing transcriptional regulators.^14^ The thermodynamic gradient from exchangeable intracellular sites to CbiK (based on the *K*_D_ of *Salmonella* CbiK for Co^II^) was calculated to result in 15% metalation of CbiK with Co^II^ (the predominant metal) in a so-called “idealized” cell, where the Co^II^ sensor (RcnR) is at the midpoint of its response range.^14^ In *E. coli* grown in LB media, aerobically or anaerobically, RcnR is well below the midpoint of its Co^II^-sensing range, which would give negligible metalation of CbiK in *E. coli*, suggesting that this may also be true in *Salmonella.*(15) However, the *K*_D_ for Co^II^ of CbiK was measured in the absence of its substrate sirohydrochlorin (SHC) and notably for the related chelatase for cofactor F_430_, CfbA, metal (Ni^II^) is partly ligated to SHC in an intermediate complex.^16^ Here, we have examined Co^II^ acquisition by CbiK in a metal-buffered *in vitro* enzymatic assay to account for such substrate binding. Co^II^ insertion by CobNST (from *Rhodobacter capsulatus*) has also been reconstituted in a metal-buffered *in vitro* enzymatic assay. The thermodynamic gradients for the flow of Co^II^ in the two biosynthetic pathways for vitamin B_12_ have thus been defined, discovering that the two chelatases require substantially (almost two orders of magnitude) different Co^II^ availabilities and uncovering the mechanistic requirement for an additional metallochaperone (CobW) in the CobNST pathway.

## Results

### Production of Reagents for Enzymatic Assays

Substrates, sirohydrochlorin (SHC) and hydrogenobyrinic acid *a*,*c*-diamide (HBAD) were synthesized *in vitro* and *in vivo*, respectively (Figure 1b). SHC was synthesized from *S*-adenosyl-l-methionine and aminolevulinic acid using copurified enzymes recovered from *E. coli* expressing tagged version of enzymes HemB and SirC from *Methanothermobacter thermautotrophicus*, HemC and HemD from *Bacillus megaterium*, and CobA from *Methanosarcina barkeri.*(18) HBAD was isolated from *E. coli* engineered to contain the set of genes (*cobAIGJFMKLHB*) for its biosynthesis.^19^ Diagnostic UV–visible absorbance spectra were recorded for both substrates (Figure 1b). The chelatases, CbiK from *Salmonella* and CobNST from *R. capsulatus*, were overexpressed and purified from *E. coli*: CbiK and CobN individually and CobST coexpressed and isolated as a complex (Figures 1b, S1 and refs (14, 20)). Metallochaperone CobW from *R. capsulatus* was overexpressed and purified from *E. coli* (Figures 1b, S1 and ref (4)).

### CobNST Metalates HBAD at Nanomolar Co^II^ Concentrations

**Figure 2:**
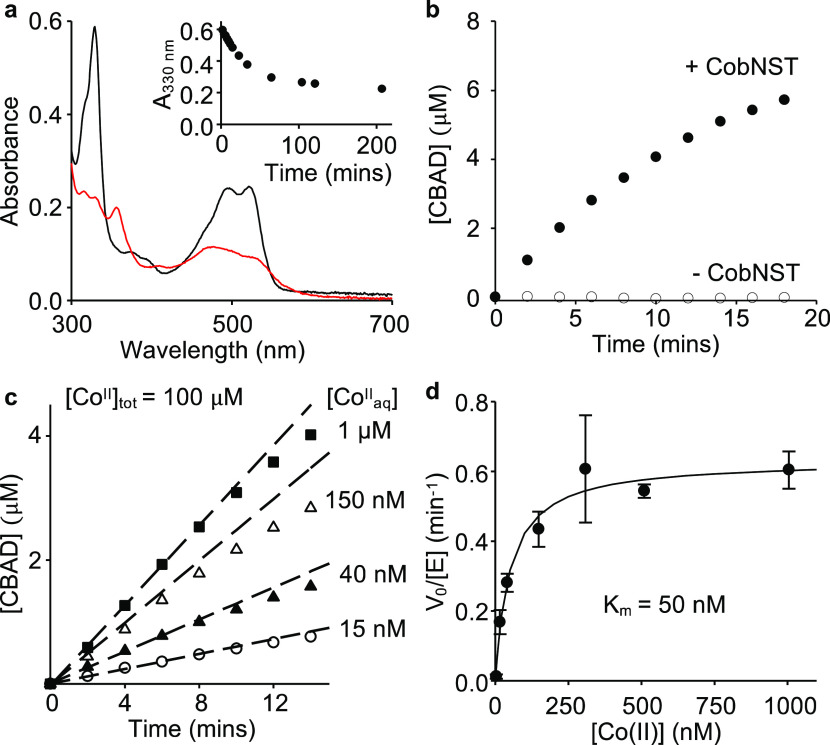
Buffered metal concentration-dependent rate of conversion of HBAD to CBAD by CobNST via steady-state kinetics. (a) UV–visible absorbance of HBAD (11.6 μM) before (black line) and 3.5 h after (red line) incubation with Co^II^ (100 μM) and CobNST (3 μM of each subunit) in the presence of MgCl_2_ (10 mM) and ATP (5 mM). The inset shows absorbance at 330 nm over time, following the addition of cobalt. (b) Initial formation of CBAD when HBAD (10 μM) was incubated with Co^II^ (100 μM), Mg^II^ (10 mM), and ATP (5 mM) in the presence (filled circles) or absence (open circles) of CobNST (3 μM of each subunit). (c) CobNST-catalyzed metalation of HBAD (initial concentration 10 μM) when available [Co_aq_^II^] was buffered to different availabilities using l-histidine to achieve sub-micromolar concentrations (Table S1). Initial rates (v_0_) were calculated from linear fits of the data for the first 6 min of reaction at each condition (shown by dashed lines). (d) Steady-state kinetics for Co^II^ insertion into HBAD by CobNST. Initial rates of metalation (v_0_) relative to enzyme concentration ([E]_tot_ = 0.5μM) were determined at varying available [Co^II^] (see Table S1 for experimental conditions). Data are the mean ± s.d. of three independent experiments. All reactions were carried out in 50 mM Hepes buffer pH 7.0, 100 mM NaCl.

The cobalt-concentration dependence of CobNST chelatase activity was determined by reconstituting the metalation reaction *in vitro*. Metalation of HBAD to form cobyrinic acid *a*,*c*-diamide (CBAD) was followed by changes in the UV–visible absorbance of the tetrapyrrole (Figure 2a), as previously reported.^9,10^ An extinction coefficient correlating absorbance change to conversion of HBAD to CBAD was determined (Δε_330 nm_ = −3.0 × 10^4^ M^–1^ cm^–1^; Figures 2a and S2). Chemical insertion of Co^II^ into HBAD was not detected in the absence of the CobNST enzyme (Figure 2b), and the rate of reaction increased proportionally with CobNST concentration, allowing reliable estimation of enzyme activity under the experimental conditions (Figure S3). To reflect intracellular metalation, avoiding artificially limiting the reaction due to the total amount of metal, available Co^II^ concentrations were controlled using ligands histidine (His) or ethyleneglycol-bis(β-aminoethyl ether)-*N*′*N*′*N*′*N*′-tetraacetic acid (EGTA) to buffer [Co_aq_^II^] from micromolar to nanomolar availabilities while retaining an excess of total [Co^II^] in each reaction mixture (Table S1). At sub-micromolar buffered Co^II^ concentrations, the enzyme-catalyzed reaction slowed significantly (Figure 2c, Table S1), following Michaelis–Menten kinetics with a fitted *k*_cat_ of 0.63 min^–1^ for metal chelation (Figure 2d), noting that similarly slow *in vitro* reaction kinetics have been estimated for the related magnesium chelatase from *Synechocystis.*(21) Importantly, the fitted *K*_m_ for Co^II^ of HBAD-CobNST is 50 nM (Figure 2d).

### Co^II^ Partitioning between CobW and CobNST

**Figure 3:**
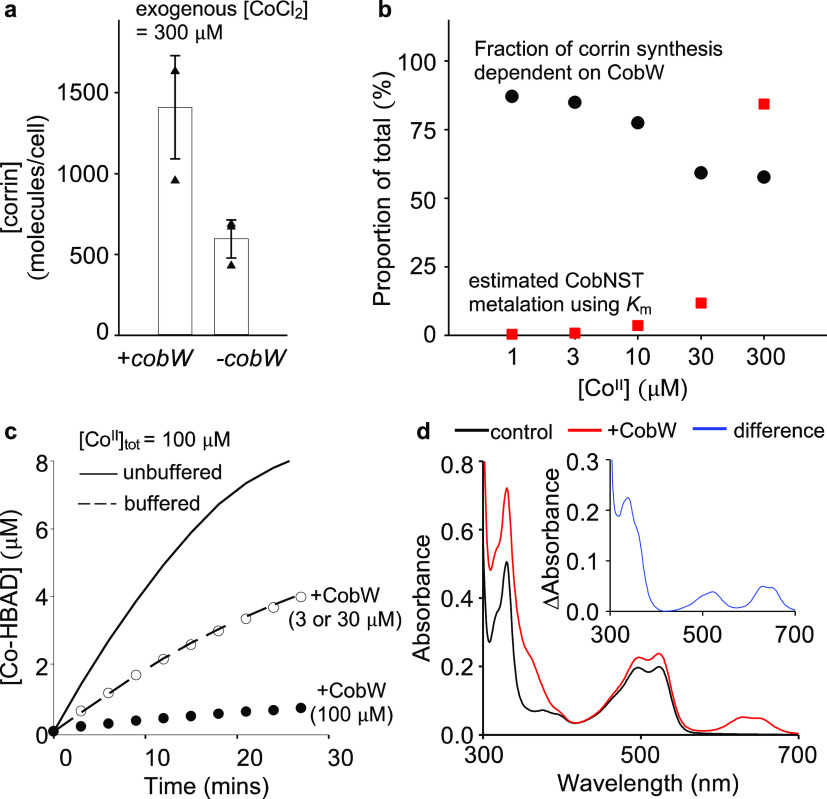
CobW enhances corrin biosynthesis *in vivo*, but Mg^II^GTP-CobW quenches the formation of CBAD by CobNST *in vitro*. (a) Corrin production by engineered E. coli* strains with and without *cobW* following 4 h exposure to 300 μM CoCl_2_. Data are the mean ± s.d. of three biologically independent replicates. Triangle shapes represent individual experiments. (b) All data refer to E. coli* cells grown in LB media with 1–300 μM CoCl_2_ supplementation. CobW-dependent corrin synthesis was determined from the difference in measured corrin production for +*cobW* versus −cobWE.coli* strains as a proportion of total corrin produced by the +*cobW* strain (panel (a) and ref (4)). CobNST metalation was estimated using *K*_m_ for Co^II^ and measured intracellular Co^II^ availabilities in E. coli* at each growth condition (Figure 2d, Table S2 and ref (4)). (c) CobNST-catalyzed formation of CBAD when Co^II^ (100 μM) was supplied in the presence of a metal buffering ligand (6 mM L-His; [Co_aq_^II^] = 40 nM) without CobW (dashed line), with 3 or 30 μM CobW (open circles, indistinguishable) or with 100 μM CobW (closed circles). The solid line shows control experiment when Co^II^ was supplied in the absence of a buffering ligand. (d) Initial absorbance spectra of reactions from (c) without CobW (black trace) or with 100 μM CobW (red trace). Difference spectra (inset) matches the known absorbance spectrum of Co^II^Mg^II^GTP-CobW with a concentration of 81 μM inferred from signal intensity at 339 nm.^4^ All reactions were performed in 50 mM Hepes pH 7.0, 100 mM NaCl with Mg^II^ (10 mM), ATP (5 mM), GTP (1 mM), and CobNST (3 μM of each subunit).

The measured *K*_m_ for Co^II^ suggests that CobNST will not be metalated at buffered intracellular Co^II^ availabilities, previously estimated as picomolar to low nanomolar concentrations in *E. coli* and *Salmonella*(4,14,15) (Table S2). Vitamin B_12_ biosynthesis was previously shown to be substantially dependent upon CobW in cultures of *E. coli* engineered to synthesize B_12_ (denoted E. coli*), grown in LB media containing up to 30 μM Co^II^ (ref (4)). Consistent with these observations, using *K*_m_ Co^II^ for CobNST, now predicts only 12% metalation of CobNST in the absence of CobW at 30 μM Co^II^ (Table S2). Cells exposed to 300 μM Co^II^ showed maximum de-repression of the *E. coli* Co^II^ sensor RcnR, which was previously used in calibrations to determine the intracellular free energies of available Co^II^ in cells grown up to 30 μM Co^II^. Figure 3a now shows CobW-dependent and CobW-independent B_12_ synthesis in cells grown in 300 μM Co^II^, revealing substantial CobW-independent synthesis at this highly elevated [Co^II^] consistent with an estimated 84% metalation of CobNST using the newly determined *K*_m_ Co^II^ (Figure 3b and Table S2). It is anticipated that the function of CobW is to make Co^II^ available to the CobNST chelatase at lower and more typical Co^II^ levels.

Intriguingly, we do still observe some (∼2-fold) CobW-dependent B_12_ synthesis even in cultures grown in LB media containing 300 μM Co^II^ where CobNST is calculated to be largely capable of independent metalation (Figure 3a,b).

To investigate the transfer of Co^II^ between the metallochaperone and chelatase *in vitro*, CobNST chelatase activity was measured at a range of available [Co^II^] (with total mol of Co^II^ in excess relative to total mol CobW), in the absence and presence of CobW, to discover whether CobW can restore enzyme activity at limiting [Co^II^] availabilities (Figure S4). Surprisingly, CobW (in the presence of its prerequisite cofactor Mg^II^GTP) had no observable effect on reaction rates when supplied in 5-fold excess of the chelatase subunits. Importantly, Figure 3c shows that, when supplied at an equimolar amount to the total amount of Co^II^, CobW quenched the chelatase activity. At such high [CobW], the absorbance spectra revealed significant changes and the difference spectra are consistent with the known spectral features of Co^II^Mg^II^GTP-CobW (Figure 3d). The increased absorbance at 339 nm, resulting from ligand to metal charge transfer (LMCT) between sulfur donors of CobW and the ligated Co^II^ ion (ε_339 nm_ = 2800 M^–1^ cm^–1^ ref (4)), indicates sequestration of most (81%) of the Co^II^ in the assay. An excess concentration of CobW (in relation to Co^II^) was sufficient to sequester all Co^II^ in the assay and fully quench the enzyme activity of CobNST (Figure S5). These data indicate that the GTP-bound form of CobW acquired Co^II^ from the reaction solution and, due to its high affinity (Mg^II^GTP-CobW *K*_Co(II)_ = 30 pM^4^), withheld metal from the chelatase inhibiting the metal insertion reaction. GTP hydrolysis weakens CobW affinity for Co^II^ (Mg^II^GDP-CobW *K*_Co(II)_ = 100 nM^4^), which should enable transfer to CobNST, implying that hydrolysis did not occur under the *in vitro* reaction conditions. Moreover, addition of GDP to the reaction mixture did not activate the chelatase reaction (Figure S4b). A GTPase-activating protein (GAP) may be missing from the *in vitro* reaction, and in direct competition, Mg^II^GTP-CobW acquires Co^II^, whereas CobNST does not as predicted from *K*_D_ versus *K*_m_.

### CbiK Metalates SHC at Sub-Nanomolar Co^II^ Concentrations

**Figure 4:**
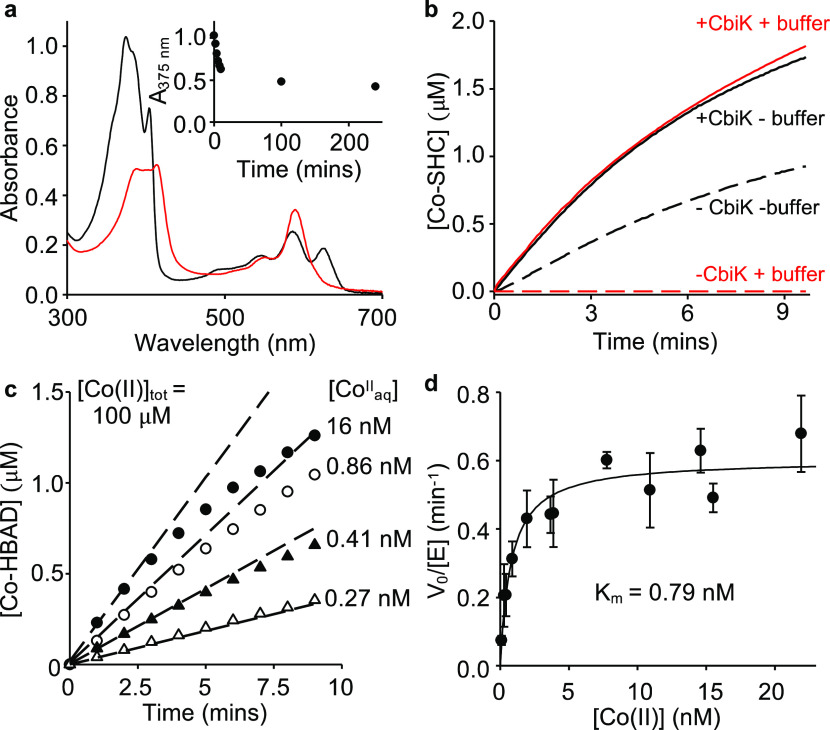
Buffered metal concentration-dependent rate of conversion of SHC to Co^II^-SHC by CbiK via steady-state kinetics. (a) UV–visible absorbance of SHC (4.3 μM) before (black line) and 4 h after (red line) incubation with Co^II^ (100 μM) and CbiK (0.5 μM) in the presence of His (1 mM). (b) Formation of Co-SHC when SHC (5 μM) was incubated with Co^II^ (100 μM) in the presence (red lines) or absence (black lines) of a histidine metal buffer (1 mM) and CbiK (0.5 μM, as labeled). (c) CbiK-catalyzed metalation of SHC (initial concentration 5 μM) when available [Co_aq_^II^] was buffered using NTA or EGTA to achieve sub-nanomolar concentrations (Table S3). Initial rates (v_0_) were calculated from linear fits of the data for the first 2 min of reaction at each condition (shown by dashed lines). (d) Steady-state kinetics for Co^II^ insertion into SHC by CbiK. Initial rates of metalation (v_0_) relative to enzyme concentration ([E]_tot_ = 0.375 μM) were determined at varying available [Co^II^] (see Table S3 for experimental conditions). Data are the mean ± s.d. of three independent experiments. All reactions were carried out in 50 mM Hepes buffer pH 7.0, 100 mM NaCl.

The Co^II^ concentration dependence of the CbiK chelatase activity from the early insertion pathway was previously explored based on the *K*_D_ of CbiK alone.^14^ To investigate whether the presence of the substrate, SHC, may enhance Co^II^ acquisition by CbiK from the intracellular milieu, metalation reactions were reconstituted *in vitro* using defined Co^II^-buffers. Co^II^ insertion into SHC was followed via changes in the UV–visible absorbance of the tetrapyrrole (Figure 4a), as previously reported.^22^ An extinction coefficient correlating absorbance change with conversion of SHC to Co^II^-SHC was determined to be Δε_330 nm_ = −1.4 × 10^5^ M^–1^ cm^–1^ (Figures 4a, S2). At excess unbuffered Co^II^ concentrations, chemical (nonenzymatic) metalation of SHC was observed, but buffering of Co^II^ to sub-micromolar availabilities prevented the nonenzymatic reaction (Figure 4b). At such buffered metal availabilities, the rate of reaction increased proportionally with CbiK concentration (within an identified set of experimental conditions, Figure S3), allowing reliable estimation of enzyme activity. Available Co^II^ concentrations were controlled using His or EGTA to buffer [Co_aq_^II^] from nanomolar to picomolar availabilities while retaining an excess of total [Co^II^] in each reaction mixture (Figure 4c, Table S3). The enzyme-catalyzed reaction followed Michaelis–Menten kinetics with a fitted v_max_ of 0.60 min^–1^ and importantly a fitted *K*_m_ for Co^II^ of 0.79 nM (Figure 4d), in contrast to a *K*_D_ of 14 nM for CbiK alone.^14^

### Responses of the *Salmonella* Sensor RcnR Used to Determine the Free Energies of Available Cobalt *In Vivo*

**Figure 5:**
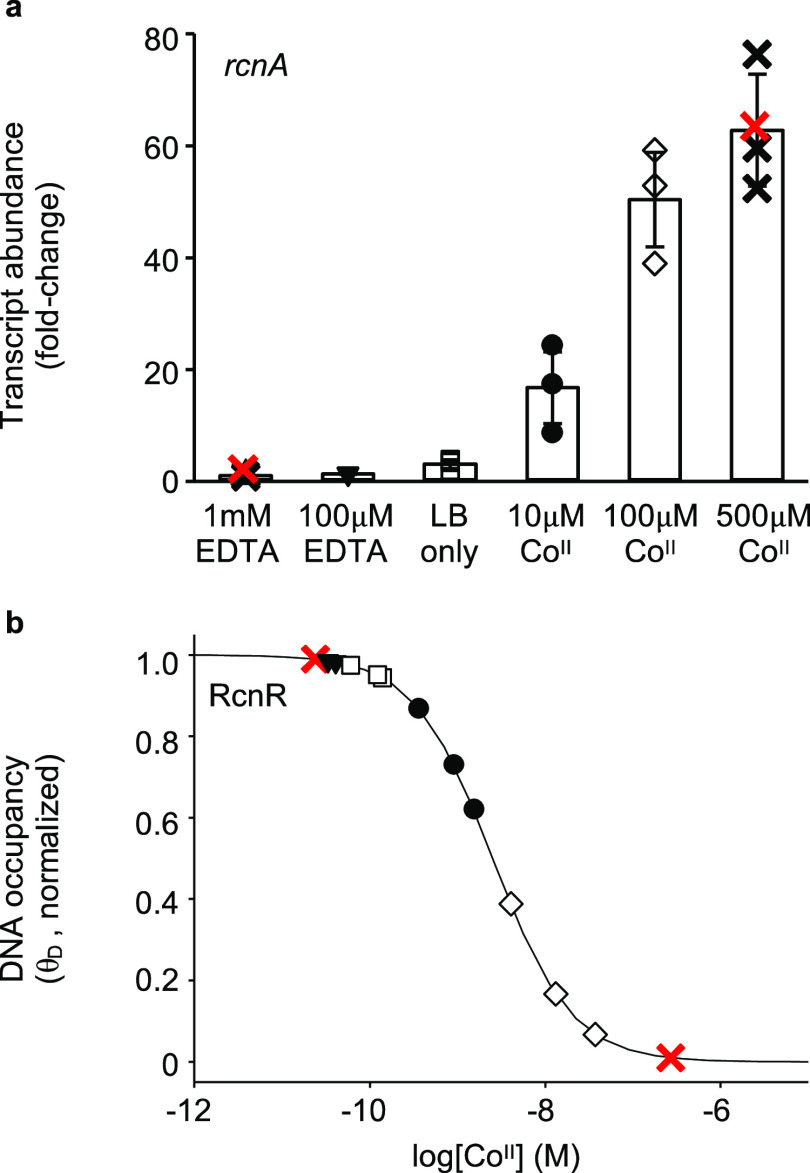
Intracellular Co^**II**^ availability in *Salmonella* under anaerobic conditions. (a) Abundance of *rcnA* transcripts (regulated by Co^II^ sensor RcnR) in anaerobic *Salmonella* cultures grown in LB media, measured by qPCR. Transcript abundances are relative to the control condition (1 mM EDTA, assigned a value of 1). Data are the mean ± s.d. of three biologically independent replicates. (b) Solid line shows the calculated relationship between intracellular available [Co^II^] and DNA occupancy of the Co^II^ sensor RcnR in *Salmonella.*(14) Fold changes in *rcnA* gene expression (from panel (a)) were converted to DNA occupancies of RcnR to determine intracellular Co^II^ availabilities for each culture. Red crosses in panel (a) indicate the minimum and maximum observed fold changes in gene expression and defined the boundary conditions (θ_D_ of 0.01 and 0.99) for the dynamic range of the sensor response in panel (b).

The >10-fold tighter *K*_m_ (relative to *K*_D_ of CbiK alone) increases estimated acquisition of Co^II^ in a cell (Figure 4d, Table S2). However, metalation is still predicted to be low in an *E. coli* cytosol (grown in LB medium, Table S2) in the absence of metal supplementation where Co^II^ availability is substantially lower than idealized *Salmonella.*(4) Notably, *E. coli* does not normally make B_12_ and lacks a dedicated Co^II^ uptake system.^23^ The CbiK protein used herein originates from *Salmonella*, an organism which makes B_12_ under anaerobic growth conditions. To estimate the metalation state of the chelatase in its native host, Co^II^ availability was measured in anaerobic *Salmonella* confirmed to be producing vitamin B_12_ (Figure S6). Cultures were grown in LB media containing varied levels of exogenous metal (by supplementing media with either CoCl_2_ or EDTA) to elicit responses from the *Salmonella* Co^II^ sensor RcnR. Changes in the abundance of the RcnR-regulated *rcnA* transcripts in isolated RNA were monitored by qPCR (Figure 5a). Cellular Co^II^ availabilities under each growth condition were determined by calibrating the responses of *Salmonella* RcnR (Figure 5b), using recently described procedures.^15^ Because Zn^II^-mismetalation of CobW had been predicted in *E. coli*,^4^ analogous experiments also determined cellular Zn^II^ availabilities in *Salmonella*, indicating that mismetalation of CobW would similarly occur in this heterologous host, albeit to a slightly lesser extent (Figure S7 and Table S5). In *Salmonella* cultured under anaerobic conditions in standard LB media, Co^II^ is more available compared to that reported for aerobic *E. coli* (Table S2). Together, tighter Co^II^ binding (*K*_m_) of CbiK in the presence of SHC and increased Co^II^ availability in anaerobic *Salmonella* enables the chelatase to become metalated in native host cells (12% predicted) substantively more than might otherwise have been estimated (Table S2).

## Discussion

**Figure 6:**
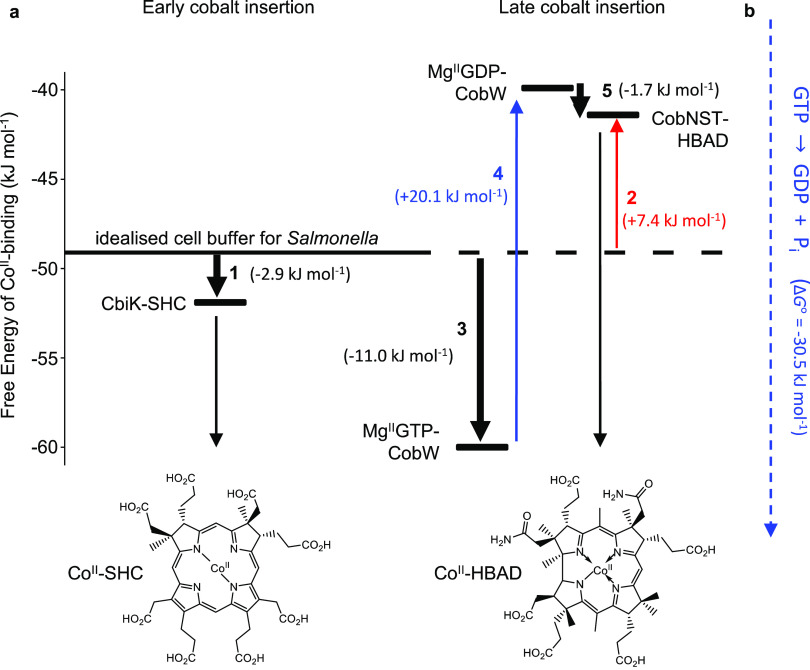
GTP-dependent CobW metallochaperone elevates the available free energy of Co^II^ binding. (a) Free energies of Co^II^-complex formation in the CbiK versus CobW/CobNST pathways (free energy calculations in Table S4). Bold arrows denote Co^II^ transfer. In the early cobalt insertion pathway, binding of the substrate SHC provides sufficient thermodynamic driving force for the chelatase CbiK to acquire Co^II^ from the intracellular milieu of idealized *Salmonella* without the need of an additional chaperone (1). Conversely, in the late cobalt insertion pathway, Co^II^ transfer from the intracellular milieu to the substrate bound chelatase, CobNST-HBAD, is thermodynamically unfavorable (2, red). Binding of Mg^II^GTP provides the necessary free energy gradient for the chaperone CobW to acquire Co^II^ in a cell (3) and nucleotide hydrolysis to generate Mg^II^GDP-CobW (4, blue) elevates the free energy of Co^II^ binding sufficiently to enable Co^II^ transfer to CobNST-HBAD (5). Representations of Co^II^-SHC and Co^II^-HBAD are placed below the scale reflective of undetermined free energy values. (b) Magnitude of the standard free energy for hydrolysis of GTP (dashed blue) is shown for comparison (note common scales but arbitrary placement of *y*-axis in panel (b)).

Figure 6 depicts the free energies for forming Co^II^-complexes with CobW (in Mg^II^GTP and Mg^II^GDP forms), CobNST (with HBAD), and CbiK (with SHC). For CbiK-SHC and CobNST-HBAD, values are free energies for forming hypothetical Co^II^-complexes that would be 50% metalated at concentrations equating to the respective *K*_m_. Since *k*_cat_ for both chelation reactions were slow (<1 min^–1^, Figures 2 and 4), *K*_m_ should approximate *K*_D_ for Co^II^ binding to CbiK-SHC and CobNST-HBAD (see eq 2). The free energy gradient for Co^II^ transfer from the intracellular milieu appears to be favorable for CbiK-SHC but unfavorable for CobNST-HBAD. This highlights the need for an accessory protein to assist cobalt acquisition in the CobNST pathway. Moreover, while the free energy gradient is highly favorable for Co^II^ transfer to Mg^II^GTP-CobW, importantly post-hydrolysis, the gradient becomes favorable for transfer from Mg^II^GDP-CobW to CobNST-HBAD. Calculations of metal transfer to and from the intracellular milieu are independent of protein concentration since the available metal is buffered. In contrast, the formation of Mg^II^GTP-CobW-CobNST-HBAD complexes would be influenced by relative protein abundances. Departure from a 1:1 (chaperone:chelatase) stoichiometry, as well as variance in measured affinities (*K*_D_ or *K*_m_), could modify the magnitude of metal transfer above or below 59% currently predicted (Calculation S1). Intermediate Co^II^-complexes at high free energy (Co^II^Mg^II^GDP-CobW and Co^II^-CobNST-HBAD) must somehow avoid transfer of Co^II^ back to the intracellular exchangeable buffering molecules (Figure 6). Insertion of Co^II^ into HBAD by CobNST is ATP-dependent, and Co^II^ becomes expeditiously trapped in the ring-contracted tetrapyrrole at this step in the biosynthetic pathway. Presumably, hydrolysis of Co^II^Mg^II^GTP-CobW occurs proximal to (or in complex with) CobNST-HBAD, consistent with a requirement for a GAP protein that is proposed to have prevented *in vitro* transfer, but it must be present in engineered *E. coli* (encoded by an introduced gene or that normally functions with native *E. coli* GTPases) (Figure 3). In this way, GTP hydrolysis, and hence the formation of the Co^II^-complexes at high free energy, can be solely restricted to clients associated with a GAP. The unfavorable free energy gradient for Co^II^ transfer from intracellular exchangeable available Co^II^ to CobNST-HBAD is calculated to be overcome by the hydrolysis of GTP mediated by CobW.

The metallochaperone-mediated transfer of Cu^I^ from hypothetical exchangeable buffer molecules to destinations (superoxide dismutase, cytochrome oxidase, and P_1_-type ATPases to the trans-Golgi network and metallothionein) follows favorable thermodynamic gradients for metal binding.^24^ This appears analogous to the CbiK-mediated transfer of Co^II^ from exchangeable buffering molecules to SHC in idealized *Salmonella*, albeit promoted by the formation of the CbiK-SHC adduct as evidenced by comparing the *K*_m_ with the *K*_D_ for Co^II^ of CbiK alone (Figures 4 and 6). Notably, the free energy for complex formation by exchangeable buffered Co^II^ is substantially lower in *Salmonella* grown in standard LB media than in idealized cells (Figure 5), estimating less than 1% Co^II^ occupancy for CbiK alone but still enabling 12% occupancy of the CbiK-SHC adduct, which may be suited to sustaining catalytic CbiK activity (Table S2). In the metallochaperone-dependent pathway, considering Co^II^ alone, 79% Co^II^ occupancy of Mg^II^GTP-CobW would be estimated in *Salmonella* grown in standard LB media (Figure 5 and Table S2), suggesting that the CobW metallochaperone may enable Co^II^ acquisition for B_12_ synthesis in organisms (including *Rhodobacter*) in which the free energy for available Co^II^ might be lower than that modeled for an idealized *Salmonella* cell. Unlike the metallochaperone-mediated delivery of Cu^I^, the CobW-dependent delivery of Co^II^ does not simply follow a favorable thermodynamic gradient for metal binding, reflecting the requirement for energetic input from nucleotide cofactors. The atypical thermodynamic pathway in Figure 6 distinguishes the CobW-dependent pathway from the Cu^I^ metallochaperone and the CbiK-dependent pathways. This also highlights metallochaperones as a mechanistically and perhaps functionally diverse grouping of proteins.

Affinities for multiple metals, coupled with intracellular availabilities revealed that Mg^II^GTP-CobW is vulnerable to mismetalation with Zn^II^ when expressed in *E. coli.*(4) Here, we calculate that Zn^II^ mismetalation of Mg^II^GTP-CobW would also be liable to occur in *Salmonella* grown in LB media (Table S5), raising questions about the relative availabilities of these two metals in the native (CobW-containing) Rhodobacter. Potentially, rates of GTP hydrolysis might be higher with Co^II^ bound rather than Zn^II^, helping to avoid any incorrect metal transfer from CobW. CbiK was previously seen to be most likely to be mismetalated by Fe^II^, albeit only 1% Fe^II^ occupancy was estimated in idealized *Salmonella* (ref^14^ and Table S5). Whether or not there would be greater Fe^II^ binding at the lesser Co^II^ availability in *Salmonella* cells grown in LB media compared to idealized cells would depend upon the *K*_m_ for Fe^II^ insertion by SHC-CbiK and the extent to which Fe^II^ availability also departs from idealized cells. Cobalt supplementation does also correlate with an increase in the total number of cobalt atoms per cell and decreases in the total number of atoms of iron (nickel and zinc) per cell (Figure S8). Notably, CbiK can also contribute to the synthesis of siroheme sufficient to partly complement cells missing the siroheme ferrochelatase CysG.^17^

Our findings suggest that it would be fruitful to explore whether or not other GTP-dependent metallochaperones enable the transfer of their cognate metals against unfavorable thermodynamic gradients. A client for the GE3 GTPase Zng1 has recently been identified as Zn^II^-requiring methionine aminopeptidase I.^25,26^ Deletion of ZNG1 in *Saccharomyces cerevisiae* impairs Map1 activity which in turn inhibits growth under Zn^II^-deficient conditions.^25^ Similarly, zebrafish and mouse mutants in Zng1 show increased sensitivity to dietary Zn^II^ starvation.^26^ It has been suggested that Map1 may in effect be unable to compete with exchangeable labile Zn^II^ buffer sites during Zn^II^ limitation and the assistance of Zng1 prioritizes the metalation of Map1 under these conditions.^25^ Bacterial cells also prioritize metalation of critical Zn^II^ clients under Zn^II^ limitation by sparing Zn^II^ demand from ribosomal subunits,^27−29^ modeled to occur when the degree of buffer saturation equates to a 100-fold decrease in available [Zn^II^] (Figure 4b from Osman and co-workers^14^). This equates to a change in free energy for metal complex formation of −11.4 kJ mol^–1^. Figure 6 shows that hydrolysis of Co^II^Mg^II^GTP-CobW elevates the free energy of Co^II^ binding by 20 kJ mol^–1^. It is plausible that Zng1 similarly acts to overcome an unfavorable thermodynamic gradient for Zn^II^ transfer from the intracellular milieu to Map1 when Zn^II^ becomes limiting. Notably, CobW still enhanced B_12_ production in 300 μM Co^II^ where CobNST is estimated to be substantially independently metalated, raising the tantalizing possibility that the metallochaperone also enhances *k*_cat_ for the enzymatic reaction in addition to overcoming an unfavorable thermodynamic gradient for Co^II^ transfer.

## Conclusions

The present work has revealed that the two pathways for vitamin B_12_ biosynthesis follow distinct thermodynamic gradients for the acquisition of Co^II^ from the intracellular milieu. While Co^II^ flows down a favorable gradient to CbiK-SHC, the G3E GTPase Mg^II^GTP-CobW can overcome an unfavorable gradient to CobNST-HBAD. Future studies should explore the intriguing implication that mechanisms exist to somehow shield hyper-available metal (at elevated Δ*G*) from solvent or nonspecific ligands to enable exclusive, tunneled/channeled transfer from GTP-dependent metallochaperones to their clients.

## Methods

### Preparation of Metal and Ligand Stock Solutions

Metal stocks (CoCl_2_, MgCl_2_ and ZnSO_4_) were prepared in ultrapure water in acid-washed glassware or clean plasticware to avoid metal contamination, with concentrations quantified by ICP-MS analysis. Ligands (NTA, EGTA, His) were prepared in ultrapure water from salts and adjusted to pH ∼7.0 prior to use in assays where necessary (i.e, when [ligand] was sufficient to affect pH of assay solution). Metal and ligand stocks were filter-sterilized before addition to bacterial cultures.

### Protein Expression and Purification

The coding regions of *R. capsulatuscobN* were cloned into a pET14b vector with a 6×His-tag and thrombin cleavage site-encoded N-terminal to the *cobN* sequence (see Figure S9 for sequence details). The coding regions of *R. capsulatuscobS* and *cobT* genes were cloned into a pET3a vector with two ribosome binding sites encoded, one immediately preceding each gene, for coexpression of the two gene products. The 6×His-tag and thrombin cleavage site included N-terminal to *cobS* only (see Figure S10 for sequence details). *E. coli* BL21(DE3) pLysS transformed with either pET14b-*cobN* or pET3a-*cobST* was cultured (with shaking) at 37 °C in LB medium with antibiotics carbenicillin (100 mg L^–1^) and chloramphenicol (34 mg L^–1^). At mid-log phase, protein expression was induced by the addition of IPTG (0.4 mM) and cells were cultured (with shaking) at 20 °C overnight. Cells were harvested and stored at −20 °C prior to use.

Cells overexpressing CobN or CobST were resuspended in 20 mM sodium phosphate pH 7.4, 500 mM NaCl, 5 mM imidazole, 5 mM DTT for lysis (sonication), and cell debris was pelleted by centrifugation (38,000*g*, 45 min, 4 °C). Lysate was loaded to a 5 mL HisTrap HP column (GE Heathcare) pre-equilibrated in suspension buffer. CobN and CobS bind to the column courtesy of the 6×His-tags at the N-terminus of each peptide, and CobT (expressed without a His-tag) remains associated with CobS throughout purification. The column was washed with suspension buffer containing 50 mM imidazole and then eluted with suspension buffer containing 300 mM imidazole. Protein-containing fractions were incubated with excess EDTA (10 mM) for ≥1 h before loading to a HiPrep 16/60 Sephacryl S-300 HR size exclusion column (Cytiva) equilibrated in chelex-treated buffer (20 mM Hepes pH 7.0, 150 mM NaCl, 5 mM DTT) and eluted in the same buffer. Peak protein-containing fractions (assessed by SDS-PAGE) were pooled, concentrated (to 0.5–1.0 mL) using a Vivaspin 15 Turbo centrifugal concentrator, and then moved to an anaerobic chamber. Concentrated samples were applied to a PD-10 desalting column prepacked with Sephadex G-25 medium (GE Healthcare) equilibrated with chelex-treated and N_2_-purged buffer (20 mM HEPES pH 7.0, 100 mM NaCl) and eluted in the same buffer. Protein purity was confirmed by SDS-PAGE (Figures 1 and S1) and concentration quantified by A_280 nm_ using extinction coefficients (ε = 134,000 M^–1^ cm^–1^ for CobN and 92,000 M^–1^ cm^–1^ for CobST) predicted from bioinformatic analysis.

Expression and isolation of *R. capsulatus* CobW and *Salmonella* CbiK followed previously reported protocols.^4,14^

### Isolation of Sirohydrochlorin

Sirohydrochlorin (SHC) synthesis and isolation followed protocols described in refs (18, 30). *E. coli* BL21(DE3)pLysS transformed with pETcoco-2ABCDC (encoding enzymes for SHC synthesis, see refs (18, 30)) was cultured (with shaking) at 37 °C in LB medium with 0.2% (w/v) glucose, carbenicillin (100 mg L^–1^), and chloramphenicol (34 mg L^–1^). At mid-log phase (OD_600 nm_ ∼0.5), 0.02% (w/v) l-arabinose was added and cells cultured a further 2 h at 37 °C; then, protein expression was induced with IPTG (0.4 mM), and cells were cultured at 24 °C overnight before harvesting pellets. Cells were resuspended in 30 mM sodium phosphate pH 7.4, 100 mM NaCl, 5 mM imidazole for lysis (sonication), and cell debris was pelleted by centrifugation (38,000*g*, 45 min, 4 °C). Lysate was loaded to a 5 mL HisTrap HP column (GE Heathcare) pre-equilibrated in suspension buffer. The column was washed with suspension buffer containing 50 mM imidazole then eluted with suspension buffer containing 300 mM imidazole. The remaining procedure was conducted in an anaerobic chamber. Peak elution fractions (∼2.5 mL) were buffer-exchanged into N_2_-purged 50 mM Tris pH 8.5, 100 mM NaCl using a Sephadex G-25 gel-filtration column, then added to solution A, and left overnight in a foil-wrapped (to exclude light) glass vessel. Solution A contained 20 mg of *S*-adenosyl-l-methionine, 10 mg of aminolevulinic acid, and 6.5 mg of NAD dissolved in 2 mL of N_2_-purged 50 mM Tris and 100 mM NaCl and adjusted to pH 8.5 using NaOH. The reaction product was applied to a 1 mL HiTrap DEAE (diethylaminoethyl) FF (GE Healthcare) pre-equilibrated in N_2_-purged reaction buffer and washed with the same buffer containing 100, 200, and 300 mM NaCl (5 mL each); then, SHC was eluted in the same buffer containing 1 M NaCl. SHC was stored frozen at −80 °C in airtight tubes and concentration quantified using ε_376 nm_ = 240,000 M^–1^ cm^–1^ ref (22).

### Isolation of Hydrogenobyrinic Acid *a*,*c*-Diamide

Hydrogenobyrinic acid a,c-diamide (HBAD) synthesis and isolation followed protocols described in ref (19), using *E. coli* strain ED549, engineered with genes for biosynthesis of hydrogenobyrinic acid from the endogenous *E. coli* precursor uroporphyrinogen III. *E. coli* strain ED549 is BL21 star(DE3)(pLysS-DNAJ^RC^-ORF647^RC^) (pETcoco2-*cob*A^*Mbar*^-*cob*IG^*Bmei*^-*cob*JFMKLH^RC^(B)^*Bmei*^) where *RC* denotes *R. capsulatus*, *Mbar* denotes *M. barkeri*, and *Bmei* denotes *Brucella melitensis*. 1L media (10 g yeast extract, 16 g tryptone, 5 g NaCl, 1 g NH_4_Cl) with ampicillin (100 mg/L) and chloramphenicol (34 mg/L) were inoculated with an overnight culture of ED549 and incubated at 28 °C with shaking (160 rpm) for 6 h before adding l-arabinose (0.2% (w/v)) and then continuing incubation at 28 °C with shaking (160 rpm) overnight. The resulting cell pellets, which contained a mixture of hydrogenobyrinic acid (HBA) and HBAD, were resuspended in 10 mM Hepes buffer pH 7.5, sonicated, and centrifuged, and the supernatant boiled for 5 min and centrifuged. The supernatant was mixed with ATP (5 mM), MgCl_2_ (20 mM), l-glutamine (5 mM), and crude extract of *E. coli* strain BL21 star(DE3)(pLysS-DNAJ^RC^-cobB^RC^) (pET3a-cobB^Bmei^), which contained overexpressed *R. capsulatus* CobB and *Brucella metlitensis* CobB for conversion of HBA to HBAD, and the mixture was left in the dark at room temperature overnight. The resulting supernatant was applied to a DEAE column pre-equilibrated in buffer (20 mM Hepes pH 7.5, 100 mM NaCl) washed with buffer containing 100 mM NaCl and HBAD eluted in buffer containing 200 mM NaCl. HBAD fractions were pooled and pH was adjusted to 4 using 1 M HCl before applying to an RP18 resin (preactivated in methanol and then rinsed with H_2_O + 0.1% TFA before applying the HBAD sample). The resin was washed with H_2_O + 0.1% TFA and then with 10% methanol, and HBAD eluted with 50% methanol. Solutions were freeze-dried and stored in the dark at −20 °C. HBAD was resolubilized in H_2_O as required and quantified using a reported extinction coefficient at λ_max_ ∼ 325 nm of ε = 50,000 cm^–1^ M^–1^ ref (9).

### Steady-State Enzyme Kinetic Assays

Reactions were prepared in an anaerobic chamber using chelex-treated, N_2_-purged 50 mM Hepes buffer pH 7.0, 100 mM NaCl and monitored spectroscopically using a sealed 1 cm path length quartz cuvette at room temperature (20–25 °C).

For CobNST enzyme assays, absorbance of the tetrapyrrole substrate HBAD in a quartz cuvette with 1.0 cm path length was monitored by continuous cycling (200–800 nm) using a Lambda 35 UV–visible spectrophotometer (PerkinElmer). Concentrations of the metalated product, cobyrinic acid a,c-diamide (CBAD), were quantified from changes in the absorbance of 330 nm, using Δε_330 nm_ = −30,000 M^–1^ cm^–1^ determined for cobalt chelation (Figures 2a and S2). The rates of metalation (v_0_) of HBAD were calculated from linear fits to changes in [Co^II^-HBAD] over the first 6 min of reactions (as shown in Figure 2c). All reactions contained [HBAD] = 10 μM, [Co^II^]_tot_ = 100 μM, [Mg^II^] = 10 mM, [ATP] = 5 mM (adjusted to pH 7.0 and quantified using ε_260 nm_ = 1.54 × 10^4^ M^–1^ cm^–1^), and [CobN] = [CobST] = 3.0 μM (based on monomeric concentrations of each subunit). Six subunits each of CobS and CobT appear to assemble a two-tiered hexameric ring, which likely docks with a single subunit of CobN to form the cobaltochelatase;^5,10^ hence, the concentration of the active CobNST enzyme was estimated to be one-sixth of the total [CobST]_monomeric_ (CobN, present at equimolar concentrations to CobST, was in excess in the reaction mixture). The available Co^II^ concentration was adjusted using buffering ligands (L-His, EGTA) as listed in Table S1. Under the experimental conditions, with an excess of Mg^II^ over ATP, the effect of ATP on free Co^II^ concentrations, and hence, the estimated *K*_m_ value for CobNST, appears to be minimal (Calculation S2). Reactions were performed in triplicate to produce a mean rate, calculated as moles of HBAD metalated per mol enzyme per min, for each available Co^II^ concentration (Table S1). The appearance of a feature at ∼350 nm is indicative of some conversion of the product to the Co^III^-form,^9^ encouraging the subsequent replacement of the oxygen detector in the anaerobic chamber.

For CbiK enzyme assays, absorbance at 375 nm was monitored in a quartz cuvette with a 1.0 cm path length using a Multiscan GO spectrophotometer (Thermo Scientific). Concentrations of metalated sirohydrochlorin produced were quantified from changes in absorbance at 375 nm, using Δε_375 nm_ = −140,000 M^–1^ cm^–1^ determined for cobalt chelation (Figures 4a and S2). The rates of metalation (v_0_) of SHC were calculated from linear fits to changes in [Co^II^-SHC] over the first 2 min of reactions (as shown in Figure 4c). Reactions contained [SHC] = 5 μM, [Co^II^]_tot_ = 100 μM, and [CbiK] = 0.375 μM. Available Co^II^ concentrations were adjusted using buffering ligands (His, NTA, and EGTA) with high concentrations of histidine ([His]_tot_ > 1 mM) avoided (see Figure S11). Ligands were added first to quench nonenzymatic metalation (see Figure 4b), and CbiK was added last to initiate the enzymatic reaction. Reactions were performed in triplicate to produce a mean rate, calculated as moles of SHC metalated per mol CbiK per min, for each available Co^II^ concentration (Table S3).

Steady-state kinetics data were fitted to eq 1
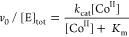
1using Microsoft Excel with fitted values for *k*_cat_ and *K*_m_ generated by minimizing the least-squares error across each dataset. This model assumes that enzymes were fully saturated with their tetrapyrrole substrates which were supplied in excess; thus, only the available [Co^II^] (buffered using ligands, see Tables S1 and S3) was rate-limiting. The Michaelis constant (*K*_m_) is described by eq 2
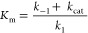
2where *k*_1_ and *k*_–1_ are the on and off rates, respectively, for Co^II^ binding to the enzyme–substrate complex and *k*_cat_ is the rate of Co^II^ insertion into the tetrapyrrole. When *k*_–1_ ≫ *k*_cat_ (i.e., metal dissociation is significantly faster than the turnover number of the enzyme), *K*_m_ approximates the  for Co^II^ binding. The presence of a large amount of buffered metal (relative to enzyme and tetrapyrrole substrate concentrations) means that the total number of Co^II^ atoms in the buffer (and hence the available [Co^II^]) did not significantly change over the course of the experiment and adventitious Co^II^ binding at nonenzymatic sites (eg His-tags on CobNST) should have negligible impact on the measured *K*_m_.

### Growth of Salmonella

All cultures and media were prepared in plasticware or acid-washed glassware to minimize trace metal contamination. For quantification of total metal, total corrin, and intracellular available Co^II^ concentrations in anaerobic B_12_-producing *Salmonella*, LB medium was inoculated with overnight culture of *S. enterica* serovar Typhimurium strain 1344 (OD_600 nm_ = 0.025) and incubated aerobically at 37 °C with shaking until OD_600 nm_ reached ∼0.5. Aliquots (30 mL) of this culture were treated with CoCl_2_, H_2_O, or EDTA (0.3 mL of 100 × concentrated stocks) to reach final concentrations specified in figure legends and then incubated statically at 37 °C in an airtight container together with Oxoid AnaeroGen anaerobic gas-generating sachets (Thermo Fisher Scientific). After 3 h of anaerobic incubation, OD_600 nm_ of each culture (Figure S12a) were recorded using a Multiscan GO spectrophotometer (Thermo Scientific), and samples for RNA extraction, quantification of corrins, and quantification of metal were collected from each culture.

For quantification of intracellular available Zn^II^ concentrations in aerobic *Salmonella*, LB medium was inoculated with overnight culture of *S. enterica* serovar Typhimurium strain 1344 (OD_600 nm_ = 0.025) and incubated aerobically at 37 °C with shaking until OD_600 nm_ reached ∼0.3. Aliquots (2.5 mL) of this culture were treated with ZnSO_4_, H_2_O, or TPEN (25 μL of 100 × concentrated stocks) to reach final concentrations specified in figure legends and then incubated at 37 °C for a further 1 h before collecting samples for RNA extraction and OD_600 nm_ (Figure S12b).

### Determination of Transcript Abundance in *Salmonella*

Samples (1 mL) of *Salmonella* cultures were stabilized in an RNAProtect Bacteria Reagent (2 mL; Qiagen) and cell pellets frozen at −80 °C for up to 1 week prior to RNA extraction. RNA was extracted using an RNeasy Mini Kit (Qiagen) as described by the manufacturer. RNA was quantified by absorbance at 260 nm and then treated with DNase I (1 U/μg RNA; Fermentas) for 1 h at 37 °C. cDNA was generated using an ImProm-II Reverse Transcriptase System (Promega) with 300 ng of RNA per reaction, and control reactions without transcriptase were conducted in parallel. Transcript abundance was determined using primers 1 and 2 for *rcnA*, 3 and 4 for *rrsD*, 5 and 6 for *rpoD*, 7 and 8 for *znuA*, and 9 and 10 for *zntA* (Table S6). Quantitative PCR analysis was carried out in 20 μL reactions using 5 ng of cDNA, 0.8 μM of the appropriate primers, and Power Up SYBR Green Master Mix (Thermo Fisher Scientific). Three technical replicates of each sample (*ie* biological replicate) were analyzed using a Rotor-Gene Q 2plex (Qiagen; Rotor-Gene-Q Pure Detection software), plus control reactions without cDNA template for each primer pair. *rpoD* was used as the reference gene in analyses of *znuA* and *zntA* transcript abundance from aerobic *Salmonella* cultures. Initial analysis of transcripts from anaerobic cultures treated with 1 mM EDTA or 500 μM Co^II^ showed significant changes in *rpoDC*_q_ values (suggesting that *rpoD* expression may have been affected by the most extreme concentrations of chelator or Co^II^) but not in *rrsDC*_q_ values which remained consistent across all samples (Table S7); thus, *rrsD* was selected as the reference gene for analyses of *rcnA* transcript abundance in anaerobic *Salmonella*. The fold change in gene abundance, relative to the mean of the control condition (defined as the condition where minimum gene transcript abundance was observed), was calculated using the 2^–ΔΔCT^ method.^31^*C*_q_ values were calculated with LinRegPCR (version 2016.1) after correcting for amplicon efficiency.^32^

### Intracellular Available **Δ***G*_metal_ under Bespoke Conditions

Fractional responses (θ_D_ or θ_DM_) of *Salmonella* metal sensors were calculated as described by Foster and co-workers^15^ using eq 3 for repressors (RcnR and Zur) and eq 4 for activators (ZntA)
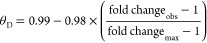
3
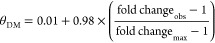
4where fold change_obs_ is the fold change in gene expression under the condition of interest and fold change_max_ is the maximum observed fold change at the calibration limits (defined as corresponding to θ_D_ or θ_DM_ of 0.01 and 0.99, respectively). Fractional responses were converted to available intracellular metal concentrations using the excel spreadsheet (Supporting Dataset 1) and MATLAB code (Supporting Note 3) from Osman and co-workers,^14^ together with known metal affinity, DNA affinities, and protein abundances determined for *Salmonella* sensors.^14^

Intracellular available Δ*G*_metal_ was calculated using eq 5
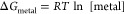
5where [metal] is the intracellular available metal concentration, *R* (gas constant) = 8.314 × 10^–3^ kJ K^–1^ mol^–1^, and *T* (temperature) = 298.15 K.

### Quantification of Total Metal and Vitamin B_12_ in *Salmonella* Cultures

Samples (10 mL) of *Salmonella* cultures were pelleted, washed twice with wash buffer (0.5M sorbitol, 200 μM EDTA, 20 mM Tris pH 8.5), and frozen at −20 °C prior to processing. The number of cells in each sample was estimated using a correlation factor previously determined for *E. coli* cells (4.4 ± 0.1 × 10^8^ cells mL^–1^ OD_600 nm_^–1^, ref (4)) to convert OD_600 nm_ to cell number. Cell pellets were resuspended in 200 μL of H_2_O, boiled for 15 min (100 °C) and centrifuged to remove cell debris. Supernatants were analyzed for total corrin and total metal.

To quantify corrin production (assumed to be predominantly B_12_ since *S. enterica* serovar Typhimurium strain 1344 contains genes for the complete pathway), an aliquot (5 μL) of each supernatant was applied to *Salmonella typhimurium* AR2680 (Δ*metE*, Δ*cbiB*) bioassay plates^33^ and incubated at 37 °C overnight. Plates were imaged on a black background GelDoc XR + gel documentation system (BioRad, ImageLab software), and diameters of growth were measured (blinded) from images. A calibration curve relating growth diameters to B_12_ concentration was generated using B_12_ standards (cyanocobalamin, 1–1000 nM, quantified by A_360 nm_ = 27,500 M^–1^ cm^–1^ at pH 10, ref (34)) in parallel with cell lysates, using the same batch of bioassay plates.

To quantify the total metal content, 100 μL of each supernatant was diluted 40-fold in 2.5% HNO_3_ (total volume = 4 mL) before metal analysis by ICP-MS.

## Supporting Information Available

Additional experimental results and calculations including rates of metalation of hydrogenobyrinic acid a,c-diamide (HBAD) by CobNST at varying cobalt availabilities (Table S1); calculated Co^II^ occupancies of proteins (CobW, CobNST, and CbiK) in bacterial cells (Table S2); rates of metalation of sirohydrochlorin (SHC) by CbiK at varying cobalt availabilities (Table S3); free energies for Co^II^ binding (Table S4); calculated Co^II^ occupancies of CobW and CbiK, taking into account competition from Fe^II^ and Zn^II^ (Table S5); oligonucleotides used in this work (Table S6); Cq values from qPCR analysis of reference genes (rpoD and rrsD) in Salmonella samples following 3 hr exposure to metal or a chelator under anaerobic conditions (Table S7); SDS-PAGE gels of proteins purified from E. coli (Figure S1); UV–visible absorbance of cobaltochelatase substrates and products (Figure S2); rates of enzymatic Co^II^ insertion into tetrapyrrole substrates as a function of total enzyme concentration (Figure S3); CobNST-catalyzed metalation of HBAD with or without CobW in the presence of surplus total Co^II^ (Figure S4); Mg^II^GTP-CobW binds and fully withholds Co^II^ from CobNST when total CobW is supplied in excess of total Co^II^ (Figure S5); quantification of vitamin B_12_ in *Salmonella* cultures (Figure S6); intracellular Zn^II^ availability in *Salmonella* (Figure S7); total metal content of S*almonella* cells (Figure S8); DNA sequence of the pET14b-CobN vector between BglII and BmtI restriction sites (including the coding region for CobN) (Figure S9); DNA sequence of the pET3a-CobST vector between BglII and SpeI restriction sites (including coding regions for CobS and CobT) (Figure S10); rates of metalation of SHC by CbiK with different Co^II^-buffering ligands (Figure S11); optical density of *Salmonella* cultures (Figure S12); calculation of Co^II^ occupancies of CobW and CobNST in the metal transfer complex (Calculation S1); and calculations to assess the influence ATP on available [Co^II^] during CobNST enzymatic assays (Calculation S2) (PDF)

## Abbreviations

ATP: adenosine triphosphate
CBAD: cobyrinic acid *a,c*-diamide
EDTA: ethylenediaminetetraacetic acid
EGTA: ethyleneglycol-bis(β-aminoether)-*N*′*N*′*N*′*N*′-tetraacetic acid
GAP: GTPase-activating protein
GTP: guanosine triphosphate
HBAD: hydrogenobyrinic acid *a,c*-diamide
His: histidine
LB: Luria-Bertani
SDS-PAGE: sodium dodecyl sulfate polyacrylamide gel electrophoresis
SHC: sirohydrochlorin
qPCR: quantitative polymerase chain reaction
