# Supplementary material for: Two Distinct Thermodynamic Gradients for Cellular Metalation of Vitamin B12
Source: JACS Au. 2023 May 10;3(5):1472–83. doi: 10.1021/jacsau.3c00119 (PMC10206600; doi:10.1021/jacsau.3c00119)
Supplement: Supplementary file 1 — au3c00119_si_001.pdf [file au3c00119_si_001.pdf]

Supporting Information

Two distinct thermodynamic gradients for cellular metalation of vitamin B_{12}

Tessa R. Young[1,2*], Evelyne Deery[3], Andrew W. Foster[1,2], Maria Alessandra Martini[1,4], Deenah Osman[1,2], Martin J. Warren[3,5], Nigel J. Robinson[1,2*]

[1] Department of Biosciences, Durham University, Durham, DH1 3LE, UK; [2] Department of Chemistry, Durham University, Durham, DH1 3LE, UK; [3] School of Biosciences, University of Kent, Canterbury, CT2 7NJ, UK; [4] Department of Inorganic Spectroscopy, Max Planck Institute for Chemical Energy Conversion, 45470 Mülheim an der Ruhr, Germany; [5] Quadram Institute Bioscience, Norwich Research Park, Norwich, NR4 7UQ, UK

E-mail: tessa.r.young@durham.ac.uk; nigel.robinson@durham.ac.uk

Table of contents

Supplementary Tables

Table S1: Rates of metalation of hydrogenobyrinic acid a,c-diamide (HBAD) by CobNST at varying cobalt availabilities

Table S2: Calculated Co^{II} occupancies of proteins (CobW, CobNST and CbiK) in bacterial cells

Table S3: Rates of metalation of sirohydrochlorin (SHC) by CbiK at varying cobalt availabilities

Table S4: Free energies for Co^{II} binding

Table S5: Calculated Co^{II} occupancies of CobW and CbiK taking into account competition from Fe^{II} and Zn^{II}

Table S6: Oligonucleotides used in this work

Table S7: C_q values from qPCR analysis of reference genes (*rpoD* and *rrsD*) in *Salmonella* samples following 3 hrs exposure to metal or chelator under anaerobic conditions

Supplementary Figures

Figure S1: SDS-PAGE gels of proteins purified from *E. coli*

Figure S2: UV-visible absorbance of cobaltochelatase substrates and products

Figure S3: Rates of enzymatic Co^{II} insertion into tetrapyrrole substrates as a function of total enzyme concentration

Figure S4: CobNST-catalysed metalation of HBAD with or without CobW in the presence of surplus total Co^{II}

Figure S5: Mg^{II}GTP-CobW binds and fully withholds Co^{II} from CobNST when total CobW is supplied in excess of total Co^{II}

Figure S6: Quantification of vitamin B_{12} in *Salmonella* cultures

Figure S7: Intracellular Zn^{II} availability in *Salmonella*

Figure S8: Total metal content of *Salmonella* cells

Figure S9: DNA sequence of pET14b-CobN vector between BglII and BmtI restriction sites (including coding region for CobN).

Figure S10: DNA sequence of pET3a-CobST vector between BglII and SpeI restriction sites (including coding regions for CobS and CobT).

Figure S11: Rates of metalation of SHC by CbiK with different Co^{II}-buffering ligands

Figure S12: Optical density of *Salmonella* cultures

Supplementary Calculations

Calculation S1: Calculation of Co^{II} occupancies of CobW and CobNST in metal-transfer complex

Calculation S2: Calculations to assess the influence ATP on available [Co^{II}] during CobNST enzymatic assays.

Supplementary References

Table S1: Rates of metalation of hydrogenobyrinic acid a,c-diamide (HBAD) by CobNST at varying cobalt availabilities[a]

[Buffer][b]	$[Co^{II}_{aq}]$ (M)[c]	$-\log[Co^{II}_{aq}]$[c]	$v_0/[E]$ (min^{-1})[d]
1 mM His	1.0×10^{-6}	6.0	0.69 ± 0.04
1.5 mM His	5.1×10^{-7}	6.3	0.59 ± 0.01
2 mM His	3.1×10^{-7}	6.5	0.67 ± 0.17
3 mM His	1.5×10^{-7}	6.8	0.46 ± 0.03
6 mM His	4.0×10^{-8}	7.4	0.31 ± 0.03
10 mM His	1.5×10^{-8}	7.8	0.19 ± 0.04
400 μM EGTA	2.6×10^{-9}	8.6	0.014 ± 0.006

[a] All reactions were performed in 50 mM Hepes pH 7.0, 100 mM NaCl with $[Co^{II}]_{tot}$ = 100 μM, [HBAD] = 10 μM, $[Mg^{II}]$ = 10 mM, [ATP] = 5 mM, [CobN] = [CobST] = 3.0 μM (equating to an active enzyme concentration of 0.5 μM, see Methods).

[b] EGTA forms a 1:1 Co^{II}:EGTA complex with $K_D = 7.9 \times 10^{-9}$ M at pH 7.0 (ref.[1]); His forms 1:1 and 1:2 Co^{II}:His complexes with $K_{A1} = 3.6 \times 10^4$ M and $K_{A2} = 1.6 \times 10^3$ M at pH 7.0, calculated as described in ref.[1] using absolute formation constants for Co^{II}-His complexes, and pK_a values for His reported in ref.[2]

[c] Denotes available free cobalt concentration.

[d] Data are mean ± S.D. of n = 3 independent experiments.

Table S2: Calculated Co^{II} occupancies of proteins (CobW, CobNST and CbiK) in bacterial cells[a]

**Growth condition**	**[Co^{II}] intracellular (M)**	**Occupancy with Co^{II}**			
		**CobW +Mg^{II}GTP [c]**	**CobNST +HBAD [d]**	**CbiK -SHC [e]**	**CbiK +SHC [f]**
***E. coli (aerobic)* [b]**					
Standard LB	2.4×10^{-11}	44 %	< 1 %	< 1 %	3 %
Hyperaerated LB	1.1×10^{-10}	79 %	< 1 %	1 %	12 %
LB + 1 µM $CoCl_2$	2.2×10^{-10}	88 %	< 1 %	2 %	22 %
LB + 3 µM $CoCl_2$	4.1×10^{-10}	93 %	1 %	3 %	34 %
LB + 10 µM $CoCl_2$	1.9×10^{-9}	98 %	4 %	12 %	71 %
LB + 30 µM $CoCl_2$	6.7×10^{-9}	> 99 %	12 %	32 %	89 %
LB + 300 µM $CoCl_2$	2.7×10^{-7}	> 99 %	84 %	95 %	> 99 %
***Salmonella (anaerobic)* [g]**					
Idealised cells	2.5×10^{-9}	99 %	5 %	15 %	76 %
Standard LB	1.1×10^{-10}	79 %	< 1 %	1 %	12 %
LB + 1mM EDTA	2.4×10^{-11}	44 %	< 1 %	< 1 %	3 %
LB + 100 µM EDTA	3.8×10^{-11}	56 %	< 1 %	< 1 %	5 %
LB + 10 µM $CoCl_2$	8.6×10^{-10}	97 %	2 %	6 %	52 %
LB + 100 µM $CoCl_2$	1.0×10^{-8}	> 99 %	17 %	42 %	93 %
LB + 500 µM $CoCl_2$	2.7×10^{-7}	> 99 %	84 %	95 %	> 99 %

[a] Calculated using equation: % occupancy = $100/(1+K_{Co(II)}/[Co^{II}])$; $K_{Co(II)}$ is the K_D (for CobW and CbiK -SHC) or K_m (for CobNST +HBAD and CbiK +SHC) with respect to Co^{II}_{aq}, [Co^{II}] is the intracellular available concentration of Co^{II} determined from the response of the cobalt sensor RcnR under specified conditions (see ref.[3] for *E. coli* and Figure 5 for *Salmonella*)

[b] Intracellular Co^{II} availabilities determined for *E. coli* cultured in standard LB media and with varying levels of $CoCl_2$ supplementation[3-5]

[c] Calculated using K_D for Co^{II} (30 pM[3]) determined for protein in association with Mg^{II}GTP.

[d] Calculated using K_m for Co^{II} (50 nM; Fig. 2) determined in the presence of HBAD.

[e] Calculated using K_D for Co^{II} (4 nM[6]) determined for protein alone.

[f] Calculated using K_m for Co^{II} (0.79 nM; Fig. 4) determined in the presence of SHC.

[g] Intracellular Co^{II} availabilities determined for idealised cells (where metal sensors are at the mid-points of their ranges)[6] or for anaerobic *Salmonella* cultured in LB media with varying levels of $CoCl_2$ or EDTA supplementation (Fig. 5).

Table S3: Rates of metalation of sirohydrochlorin (SHC) by CbiK at varying cobalt availabilities[a]

**[Buffer]**[b]	**[Co^{II}_{aq}] (M)**[c]	**-log[Co^{II}_{aq}]**[c]	$v_0/[E]$ **(min^{-1})**[d]
200 μM NTA	2.2×10^{-8}	7.7	0.68 ± 0.11
250 μM NTA	1.5×10^{-8}	7.8	0.63 ± 0.06
150 μM EGTA	1.6×10^{-8}	7.8	0.49 ± 0.04
300 μM NTA	1.1×10^{-8}	8.0	0.51 ± 0.11
200 μM EGTA	7.8×10^{-9}	8.1	0.60 ± 0.02
300 μM EGTA	3.9×10^{-9}	8.4	0.45 ± 0.10
700 μM NTA	3.7×10^{-9}	8.4	0.44 ± 0.05
500 μM EGTA	1.9×10^{-9}	8.7	0.43 ± 0.08
1 mM EGTA	8.6×10^{-10}	9.1	0.31 ± 0.05
2 mM EGTA	4.1×10^{-10}	9.4	0.21 ± 0.06
3 mM EGTA	2.7×10^{-10}	9.6	0.21 ± 0.09
10 mM EGTA	7.8×10^{-11}	10.1	0.07 ± 0.01

[a]All reactions were performed in 50 mM Hepes pH 7.0, 100 mM NaCl with $[Co^{II}]_{tot}$ = 100 μM, [SHC] = 5 μM, [CbiK] = 0.375 μM.
[b]EGTA and NTA form 1:1 Co^{II}:ligand complexes: at pH 7.0 EGTA $K_D = 7.9 \times 10^{-9}$ M (ref.[1]); and NTA $K_{Co(II)} = 2.2 \times 10^{-8}$ M (ref.[1]).
[c]Denotes available free cobalt concentration.
[d]Data are mean ± S.D. of n = 3 independent experiments.

Table S4: Free energies for Co^{II}-binding

	K_D or K_m for Co^{II} (M)[a]	$\Delta G_{Co(II)}$ (kJ mol^{-1})[b]
Intracellular buffer	2.5×10^{-9}	-49.1
CbiK	1.4×10^{-8}	-44.8
CbiK-SHC	7.9×10^{-10}	-51.9
CobNST-HBAD	5.0×10^{-8}	-41.7
Mg^{II}GTP-CobW	3.0×10^{-11}	-60.0
Mg^{II}GDP-CobW	1.0×10^{-7}	-39.9

[a]K_D was measured for CbiK alone (ref.[6]) and for nucleotide-bound complexes of CobW (ref.[3]). K_m was measured for CobNST-HBAD and CbiK-SHC (Figure 2 and Figure 4, respectively). Intracellular buffered metal availability is based on idealised *Salmonella* cells (ref.[6]).
[b]$\Delta G = RT\ln(K)$, where $K = K_D$ or K_m

Table S5: Calculated Co^{II} occupancies of CobW and CbiK taking into account competition from Fe^{II} and Zn^{II} [a]

Growth condition	Metalation		
	**CobW +Mg^{II}GTP** [b]	**CbiK -SHC** [c]	**CbiK +SHC** [d]
Idealised cells [e] (*Salmonella*)	92 % Co^{II}<br>7 % Zn^{II}	15 % Co^{II}<br>1 % Fe^{II}	76 % Co^{II}<br>1 % Fe^{II}
Salmonella LB [f]	25 % Co^{II}<br>68 % Zn^{II}	n/a	n/a
E. coli LB [g]	18 % Co^{II}<br>74 % Zn^{II}	1 % Co^{II}<br>29 % Fe^{II}	6 % Co^{II}<br>27 % Fe^{II}

[a] Using metalation calculators which consider inter-metal competition[3, 4]. Calculations allowed for Co^{II} and Zn^{II} binding for Mg^{II}GTP-CobW, and for Fe^{II} and Co^{II} binding for CbiK.
[b] Calculated using K_D for Co^{II} and Zn^{II} binding to Mg^{II}GTP-CobW [3].
[c] Calculated using K_D for Co^{II} and Fe^{II} binding to CbiK alone[6].
[d] Calculated using K_m for Co^{II} binding to CbiK-SHC (Figure 4), and K_D for Fe^{II} binding to CbiK alone[6]
[e] Using intracellular metal availabilities for idealised *Salmonella* cells determined from mid-point of metal sensor responses[6].
[f] Using intracellular Co^{II} availabilities determined for *Salmonella* cells cultured anaerobically in LB (Figure 5 and Supplementary Table 2), and Zn^{II} availabilities for *Salmonella* cultured aerobically in LB (Supplementary Figure 7).
[g] Using intracellular metal availabilities determined for *E. coli* cells cultured aerobically in LB[4].

Table S6: Oligonucleotides used in this work

No.	Primer Name	Sequence	Reference
1	rcnA_RTPCR_F	5′-TCCCAGCGCCATTTTATTAG-3′	ref.[7]
2	rcnA_RTPCR_R	5′-ACGATCGCGGTATGAGAAAG-3′	ref.[7]
3	rrsD_RTPCR_F	5′-GCACAACCTCCAAGTAGACATC-3′	ref.[7]
4	rrsD_RTPCR_R	5′-GGTGAAATGCGTAGAGATCTGG-3′	ref.[7]
5	rpoD_RTPCR_R	5′-CAACCGTATTTCTCGCCAGATG-3′	ref.[7]
6	rpoD_RTPCR_R	5′-CACCCAGATGCGAATCTTCATC-3′	ref.[7]
7	znuA_RTPCR_F	5′-ACATGCATCTTTGGCTCTCC-3′	ref.[7]
8	znuA_RTPCR_R	5′-ACCGACCTGTTTATCGGTTG-3′	ref.[7]
9	zntA_RTPCR_F	5′-TAAACTGGTTTCCGGTTTCG-3′	ref.[7]
10	zntA_RTPCR_R	5′-TCAATCAGCGTCAGGATACG-3′	ref.[7]

Table S7: C_q values from qPCR analysis of reference genes (*rpoD* and *rrsD*) in *Salmonella* samples following 3 hrs exposure to metal or chelator under anaerobic conditions[a]

Condition	C_q *rpoD*	C_q *rrsD*
1 mM EDTA	27.5	9.4
100 μM EDTA	26.3	8.9
untreated	26.3	9.2
100 μM $CoCl_2$	26.4	9.5
500 μM $CoCl_2$	24.9	9.2

[a] Data from a single preliminary biological analysis suggesting metal-dependent variation in *rpoD* but not *rrsD*

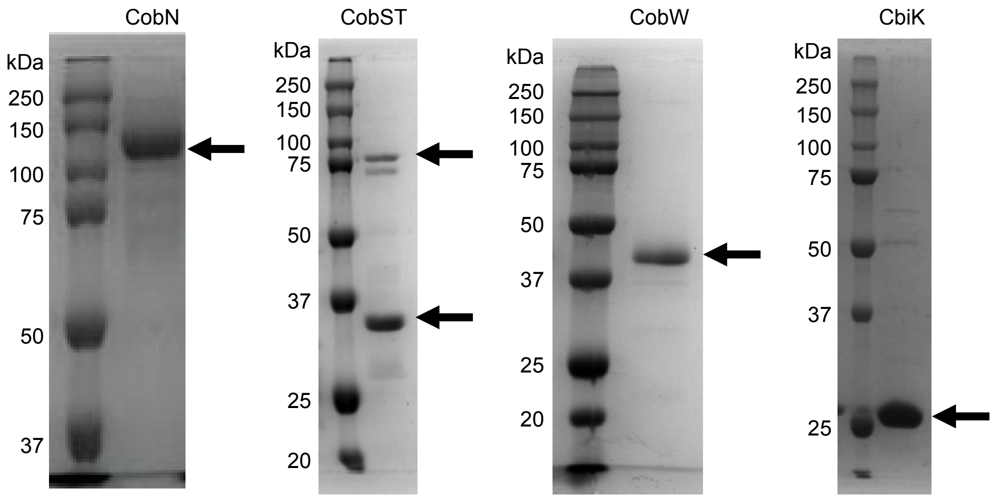

Figure S1: SDS-PAGE gels of proteins purified from *E. coli*

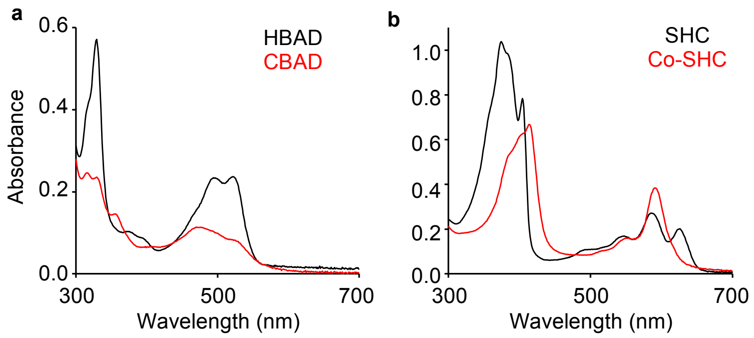

Figure S2: UV-visible absorbance of cobaltochelatase substrates and products

a UV-visible absorbance of HBAD (11.4 μM) before (black line) and after (red line) prolonged incubation (95 mins) with Co^{II} (100 μM) and CobNST (3 μM of each subunit) in the presence of $MgCl_2$ (10 mM) and ATP (5 mM). Extinction coefficient of $\Delta\varepsilon_{330\ nm}$ = -30,000 M^{-1} cm^{-1} for the transformation of HBAD to CBAD is the mean of two spectra (also Fig 1b). **b** UV-visible absorbance of SHC (4.3 μM) before (black line) and after (red line) prolonged incubation (overnight) with Co^{II} (100 μM) and CbiK (0.5 μM). Extinction coefficient of $\Delta\varepsilon_{375\ nm}$ = -140,000 M^{-1} cm^{-1} for the transformation of SHC to Co-SHC is the mean of two spectra (also Fig 3b).

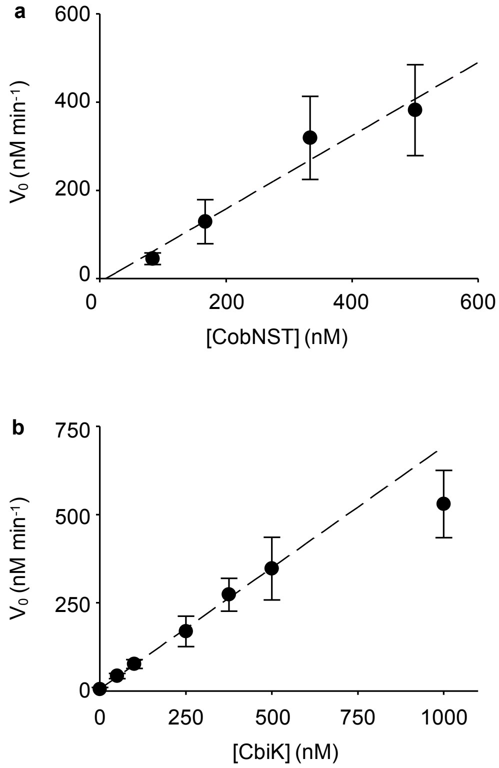

Figure S3: Rates of enzymatic Co^{II} insertion into tetrapyrrole substrates as a function of total enzyme concentration

a Conversion of HBAD to CBAD catalysed by CobNST ($[HBAD]_{initial}$ = 10 μM; reaction solution also contained $[MgCl_2]$ = 10 mM and [ATP] = 5 mM); **b** Conversion of SHC to Co^{II}-SHC catalysed by CbiK ($[SHC]_{initial}$ = 5 μM). Data are the mean ± s.d. of n = 3 replicates. All reactions were performed in 50 mM Hepes buffer pH 7.0, 100 mM NaCl. Reactions involving SHC also contained 1 mM His to suppress non-enzymatic Co^{II} insertion (see Fig. 3b). Under the given experimental conditions the rate of reaction in **a** increased proportionally with [CobNST] for all enzyme concentrations tested (NB: concentrations of active enzyme were calculated as one sixth of the total concentration of CobST subunits, see Methods); and the rate of reaction in **b** increased proportionally with [CbiK] for enzyme concentrations $\leq$ 500 nM.

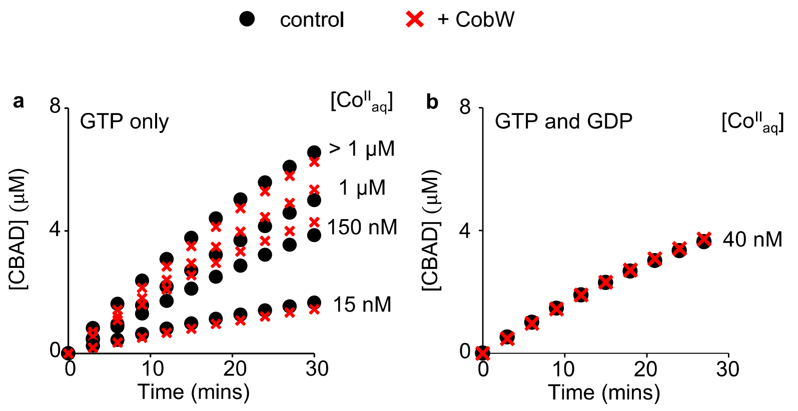

Figure S4: CobNST-catalysed metalation of HBAD with or without CobW in the presence of surplus total Co^{II}

a Formation of CBAD over time when HBAD (10 μM) was incubated with Co^{II} (100 μM), CobNST (3 μM of each subunit), Mg^{II} (2.7 mM), ATP (5 mM) and GTP (1 mM) in the absence (black circles) or presence (red crosses) of CobW (15 μM). Available $[Co^{II}_{aq}]$ in solution were controlled (from 15 – 1000 nM) using L-His as a metal buffer (see Supplementary Table 3), reactions without addition of a metal buffer ($[Co^{II}_{aq}]$ > 1 μM) also shown for comparison. **b** As in **a** with [CobW] = 30 μM and addition of GDP (1 mM) to each reaction. All reactions performed in 50 mM Hepes buffer pH 7.0 100 mM NaCl. CobW had no observable effect on HBAD metalation in these *in vitro* reaction setups.

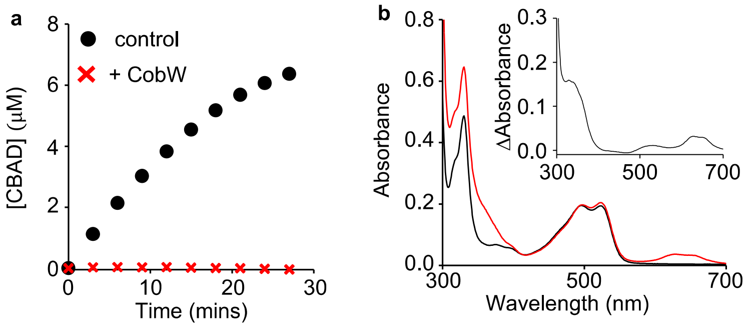

Figure S5: Mg^{II}GTP-CobW binds and fully withholds Co^{II} from CobNST when total CobW is supplied in excess of total Co^{II}

a Formation of CBAD over time when HBAD (10 μM) was incubated with Co^{II} (50 μM), CobNST (3 μM of each subunit), Mg^{II} (2.7 mM), ATP (5 mM) and GTP (1 mM) in the absence (black circles) or presence (red crosses) of CobW (120 μM). **b** Absorbance of reactions from **a** immediately following mixing of reactants. Inset shows difference spectra and corresponds to the known absorbance spectrum of $Co^{II}Mg^{II}$GTP-CobW (estimated concentration from $A_{339\ nm}$ ~ 55 μM, see ref.[3]). Data indicates that solution Co^{II} has been fully sequestered by Mg^{II}GTP-CobW, preventing metal acquisition and formation of CBAD by CobNST.

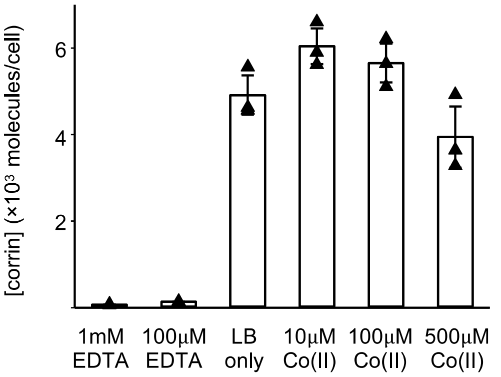

Figure S6: Quantification of vitamin B_{12} in *Salmonella* cultures.
Total corrin concentration in *Salmonella* cells grown in LB media following 3 hr anaerobic exposure to EDTA or $CoCl_2$, determined via bioassay[8]. Data are the mean ± s.d. of n=3 biological replicates (black triangles show individual experiments).

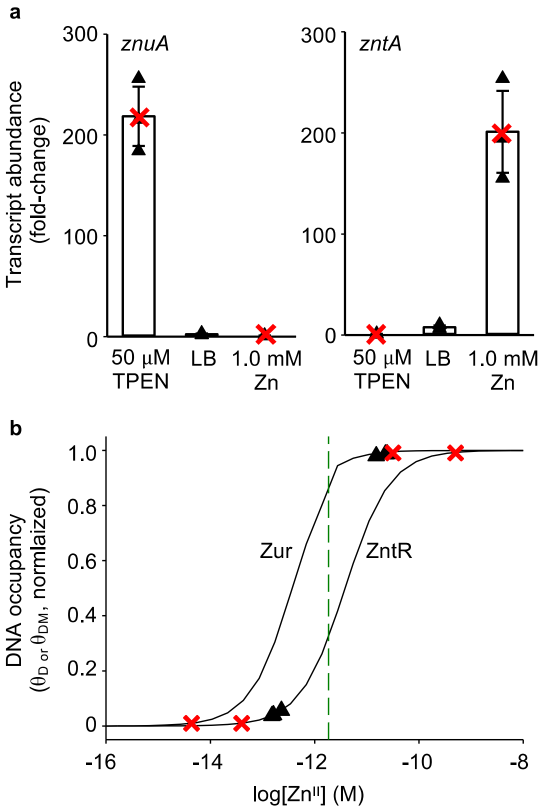

Figure S7: Intracellular Zn^{II} availability in *Salmonella*

a Abundance of *znuA* and *zntA* transcripts (regulated by Zn^{II} sensors Zur and ZntR, respectively) in *Salmonella* cultures grown in LB media, measured by qPCR. Transcript abundances are relative to control condition where lowest gene expression was observed (1 mM Zn for *znuA* and 50 µM TPEN for *zntA*, assigned values of 1). Data are the mean $\pm$ s.d. of 3 biologically independent replicates. Triangle shapes represent individual experiments. Cells were cultured under aerobic conditions, and it is noted Zn^{II} availabilities in *E. coli* were similar in aerobic and anaerobic conditions[4]. **b** Solid line shows the calculated relationship between intracellular available [Zn^{II}] and DNA occupancy for Zn^{II} sensors Zur and ZntR in *Salmonella*[6]. Fold-changes in gene expression (from panel **a**) were converted to DNA occupancies of sensors to determine intracellular Zn^{II} availabilities for each culture (black triangles). The red crosses in **a** indicate the minimum and maximum observed fold-changes in gene-expression and defined the boundary conditions (θ_D of 0.01 and 0.99) for the dynamic ranges of sensor responses in **b**. The intracellular Zn^{II} availability in *Salmonella* grown in LB media, calculated from the mid-point between mean measured responses for Zur and ZntR, is 1.85×10^{-12} M (green dotted line).

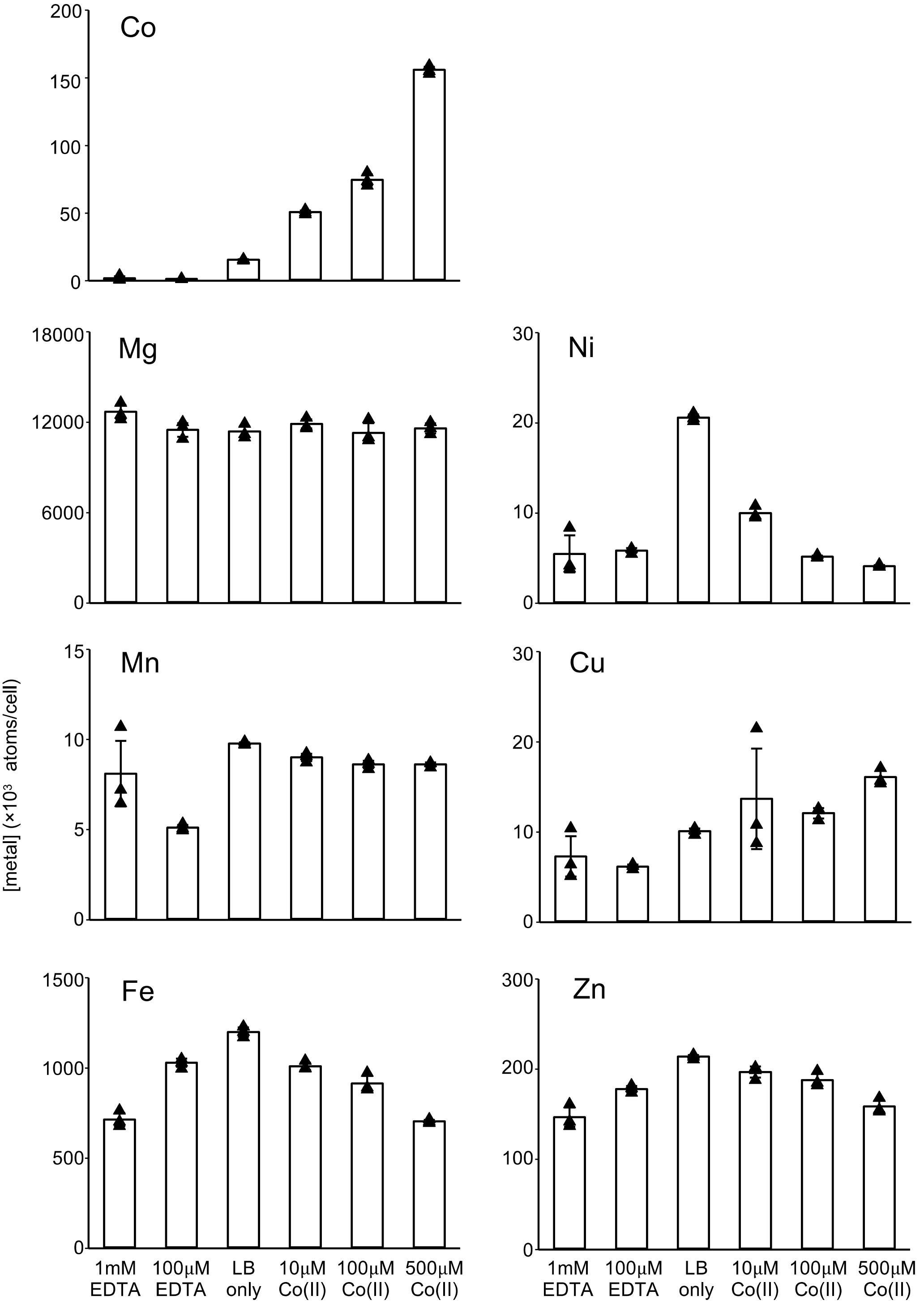

Figure S8: Total metal content of *Salmonella* cells.
Total concentration of Co, Mg, Mn, Fe, Ni, Cu and Zn in $CoCl_2$- or EDTA-treated *Salmonella* cells was determined by ICP-MS. Data are the mean ± s.d. of n=3 biological replicates (triangles show individual experiments).

AGATCTCGATCCCGCGAAATTAATACGACTCACTATAGGGAGACCACAACGGTTTCCCTCTAGAAATAATTTTGTTTAACT
TTAAGAAGGAGATATACCATGGGCAGCAGCCATCATCATCATCATCACAGCAGCGGCCTGGTGCCGCGCGGCAGCCATAAT
ATGCATGTCGTCTTCCGCGAAAGCCACGGGCTCGAGGAAACCGCGGTGCCGACCGATCTGGGTCAGCGCCCGGCGGATCTT
GTGGTGCTGTCGCTCTCTGACAGCGATCTGGGGGCTTTCGCGGCGGGCTGGAAACGCGCGGGCGGGCGGCTGCCCTCGCTG
CGGCTGGCCAATCTGGTGGCGCTGAAACATCCGATTTCGGTCGATACTTACGTTGAAAACACGCTGTCGGGGGCGAAGGGG
ATCCTGATCCGGCTGATCGGCGGCGAGGCTTACTGGCCCTATGGTCTGGCCTCGGTGCAGGATCTGTGCCGCCGCAAGGGG
ATTGCCTTGGCCGTCCTTCCCGCCGATGGCCGCGCCGATGACCGGCTCGATGCGCTCTCGACCCTGCCGGTTTCGACGCTG
CGGCGGCTCAAGGAGCTTTGCGATGCGGGCGGCGCGGTGGCGGCGCAGGCGGCCTTGGCGCAGCTGGCCCTGGCCTCCGGC
CTTTACGCCCCCCCGGTGCCGGGCGACAAGCGCCTGCCCGACTGGGGGTTTTACGACCCGGCCGCGGGCGTGATCCCCGCG
CCCGAGGGGGACGGCCCCCTGGCGCTGGTCAGCTTCTACCGCTCTTATCTGACCGCCGCCGATACCGCGCCGATCGATGCG
CTGATCCATGGCCTGCGCGCGCGCGGTTTCGCGGCTTGCGGCCTCTTTGCGCCCTCGCTCAAATCCGAGGGGCTGGGCGAT
GCGCTGCAAGCCCGGCTGGACGGCCGCGCCCCCGCGCTCGTCGTCAACGCGACCGCCTTTTCGGCGCAGGGCGCGGATGGC
ACGACGCCCTTTGACCGCTATGATGTGCCGGTGTTTCAGGTCGCGCTCTCCACCGCCCGCAAGCGCGACTGGGCCTTGGCC
GACCGCGGCCTTTCGCCTGCCGATCTGGCCATGCATGTCGTGCTGCCCGAGGTCGATGGCCGTCTGTTCGCCGGTGTCGTC
TCCTTCAAGGCGCCCGAGCCGCGCGACCCCGATCTGGAATTTTCCCGCTTCGCCCATCGCCCCGATCCGGCGCGGGTCGAG
GCCGCGCTGGACCGGATCTGCGGCTGGCACCGCTTGCGGCAAACCCCGGCGGCGGATCGCAAGCTGGCGGTGGTGCTGTCC
ACCTATCCCGGCCGGTCCTGGCAGATGGCCCATGCGGTGGGGCTGGATGCGCTCGCCTCGACCGAGGCTTTGCTGGCCCAG
CTTGCGACCGAGGGCTATGCGATTGCCCCCGGTGCCGCACTTGAGACACTGACGACCGCGCGTCTGACCTGGCCGCTTTCG
GATTATCTCACCGCGCTGGACACCCTGCCTGAAAAGCTGCGCGCCGATCTGACCGCGGCCTGGGGGCCGCCCGAGGCTGAC
CCCGCCTGCGCCGACGGTGCCTTTCACTTCGCCGCCCTGCGCCGCGGCGCCGCGCTGATCGCGCTGCAGCCCGAACGCGGC
GATGTGCAAACCCGCGACGGCGAGTATCACGACCTGTCCCGCGTGCCGCGGCATGGCTATGTCGCCTTTTATCTCTGGCTG
CGGGCCTTGGGCCTCGACGCGCTGGTGCATATGGGCGCGCATGGCACGCTCGAATGGCTGCCGGGCAAGGCCGTTGCGCTG
TCGGAAAGCTGCTGGCCTGAGGCGCTTTGCCCGCTGCCCGTCATTTATCCCTTTATCGTCAATGACCCGGGCGAGGCGGCG
CAGGCGAAACGGCGCCTTTCGGCCGTGACTTTGGGCCATCTGCCGCCGCCCCTGTCGCAAGCCGCCGTGCCCGAGGGGATG
GCGCGGCTCGAACGGCTTTTGGACGAATATTCCACCGCCGACGGGTTGGACCCGGCCCGGCGCGACCGGCTGATCGCCGAC
ATCCGCGACGAGGCCCGCGCGAGCGGCGTCGAAGACGATCTGGGCATTCCCGCCACCGCCTCGGCCGCGGAAGCGATCACC
CGCATCGACCGTTTTGTCTGCGATATCAAGGAAAGCCTGTTTGGCGACGGGCTGCATGTGCTGGGGCAGGGCGCCTGCGGG
GCTGCCGAACAGGCGGGGCTTTCGGCGGCCTTGGCGGGCAAACGCGTGCCCCCCGGCCCCTCGGGCAGCCCGGCGCGCGGG
CGCGCGGACGTGCTGCCGACCGGGCGCAACCTGTTCTCCGTCGATCCCCGGGCGGTGCCCAGCCGTGCCGCCCATGCCCAA
GGCGTGAAGCTGGCCGAGGAGCTGCTTCGAAAACATCTGCAGGATCATGGCGACTGGCCGCGCGGTCTGGTCGTCGATCTT
TGGGGCTCGGCCACGATGCGCACCGCGGGCGAGGAATTCGCCATGGCGCTGCATCTGGCCGGTCTGAAACCGGTCTGGGAC
GAGGGCGCCGCCCGGGTTTCGGGGGTCGAGGTGATCGCGCTTGCGCTTCTGGGCCGCCCACGCATCGACGTGACCCTGCGC
GTTTCGGGCCTTTTCCGCGATATCTTCCCCGGCCTTGCGCAGCTCTTCGAGACCGGCGCCGAGGCGCTGGCCACGCGCGAG
GAAACGCCCGAGGACAACCCCTACCGGGCTGCGGCCCCGCGCGTCTTCGGGCCGCGTCCCGGCCAATACGGGCTGAACATG
GGCCCGGCGCTGGAGGCCTTTACCCCCGAAGCCCGCGCAAGGGCCGGCGAGGCCTGGCTGGCGGCCTCCTCCTACACGATT
GACAAGAAAGGCGAAATCCGCTCCGACCGGGCGGGGCTGGAAGCCCGCCTGATGGGGGCCGACAGTTTCGTGCATGCCCAG
GATCTGCCCGAAAGCGATATCCTGCTGGCCTCGGATTACGCCGCGCATGAGGCCGGGTTTGCCGCCGCCCTTGCGCGTCTG
GGCGCCGAGGCCCCGGCGCTTTATCATCTGGACGCCACCGACCCGACCCGGCCCCGCGCCCGCTCGCTGCCCGAGGAGATC
GCCCGCGTCGTGCGCGCGCGCGCCGCGAACCCGGCCTGGGCCGATGGGATGATGCGGCACGGCTTCCGCGGCGGGGCGGAA
ATCGCCGCGACGCTCGACCACATGGCGGCTTTCGCGCATCTGGCCCAAGTCGTGCCCGCGCATCTGTTCGACCTTTACCAC
GAGGCGACGCTGGGCCGCGACGAGGTGCGCGACTTTCTGGCCCGCGAAAACCCCGAGGCGCTGGCGGCGATGCGCGATCTG
TTTCAACGCCTGGCCGAGGCGGGCCTCTGGCTGACGCGGCGCAATTCGATTGCGGCAAGCCTCGAGGAAAGGCTAGAAGCG
CGCCAACCCGAAGGATGACTAGTTTTGGATCCGGCTGCTAACAAAGCCCGAAAGGAAGCTGAGTTGGCTGCTGCCACCGCT
GAGCAATAACTAGCATAACCCCTTGGGGCCTCTAAACGGGTCTTGAGGGGTTTTTTGCTGAAAGGAGGAACTATATCCGGA
TATCCACAGGACGGGTGTGGTCGCCATGATCGCGTAGTCGATAGTGGCTCCAAGTAGCGAAGCGAGCAGGACTGGGCGGCG
GCCAAAGCGGTCGGACAGTGCTCCGAGAACGGGTGCGCATAGAAATTGCATCAACGCATATAGCGCTAGC

Figure S9: DNA sequence of pET14b-CobN vector between BglII and BmtI restriction sites (including coding region for CobN highlighted grey).

AGATCTCGATCCCGCGAAATTAATACGACTCACTATAGGGAGACCACAACGGTTTCCCTCTAGAAATAATTTTGTTTAACT
TTAAGAAGGAGATATACATATGCTCGACCAGACCGCCAAGCCCACCGAAGAGATCGATCTGCGCGAAGTCTTCGGGATCGA
CAGCGACATGAAGGTGAAGGCCTTTGCCGAACGCACCGACCGGGTGCCGGACCTTGACCCGACCTACAAATTCGACCCCGA
CACCACGATGGCGATTCTGGCAGGCTTTGCCTACAACCGCCGCGTGATGATTCAGGGCTATCACGGCACCGGCAAATCGAC
CCATATCGAACAGGTGGCGGCGCGGTTGAACTGGCCCTGCGTGCGGGTGAACCTTGACAGCCACGTGAGCCGGATCGACCT
GATCGGCAAGGATGCGATCAAGCTCGTTGACGGCAAGCAGGTCACGGTCTTCCACGAGGGGATTCTGCCCTGGGCGCTGCG
CAACCCGACCGCGATTGTGTTCGATGAATACGACGCCGGCCGCGCCGATGTGATGTTCGTGATCCAGCGCGTTCTGGAAGC
CGACGGGAAACTGACGCTGCTCGACCAGAACGAAGTGATCACCCCGAACCCCTATTTCCGGCTGTTCGCGACGGCGAACAC
CGTGGGTCTGGGCGACACGACCGGGCTTTATCACGGCACGCAGCAGATCAACCAGGGCCAGATGGACCGCTGGTCGCTTGT
CGCGACGCTGAACTATCTGTCCCACGACGCCGAGGCCGCGATCGTCCTCTCGAAAGTGCCGCATTACAACACCGACAAGGG
CCGCAAGGTGATCAACCAGATGGTGACCGTGGCCGATCTGACCCGGACCGCCTTCATGGCCGGGAACCTCTCGACCGTGAT
GTCGCCCCGCACCGTCATCGCCTGGGCGCAGAACGCCGATATCTTCCGCAACGTGGGCTATGCTTTCCGGCTGACCTTCCT
GAACAAATGCGACGAACTCGAACGCGCCACCGTGGCCGAGTTCTACCAGCGTCTGTTCGACGAGGAACTGCCCGAAAGCGC
CGCCGTCATCGCGGGGCGCTAAGGGGGGGCGATGACGAAGCCCACTGACGGATCCGAAACTAGAAATAATTTTGTTTAACT
TTAAGAAGGAGATATACCATGGGCAGCAGCCATCATCATCATCATCACAGCAGCGGCCTGGTGCCGCGCGGCAGCCATATG
ACGAAGCCCACTGACAACCCCGCCGATCCGTTCAAGAAGGCCCTGGCCGAGGCCACCCGGACCTTGGCCGATGCACCCGAA
CTGAGCGTCAGTTTCAGCGTCGATCCGCCCGGTGTGGCGGGCGATCAGATGCGCCTGCCGCAGGTCTCGCGCCGGATGACG
CGCGACGAGATCATGATGGCGCGCGGCACCGGCGATGCTTTGGCCCTGCGCCAGCGTCACCACGATACCGCCGTGGCCGCC
CGCTATGCGCCCTCGGGCGACATGGCGAAATCGCTTTACGAGGCGATGGAAACCGCCCGCTGCGAGGCTTTGGGCGCGCGC
GACATGCCCGGCACGCTGACCAATATCGACTTCAAGATCGGCCATGAGGCCGAGCGCAAGGGCTATGGCCAGATCCGCACC
CAGGCCGAGGCGCCGCTTGCGGTGGCCGCGGGCTATCTGGTGCGCGAAATGGCGACGGGGCGGAAACTGCCGCAGGCCGCG
AACCATGTGCTCGACCTCTGGCGCGGCTTTCTTGAGGATCAGGCGGGCGAGGAGTTTCAGGGCATCGAGGCGGCTCTGGCC
GATCAGGCGGCCTTTGCACGTCTTGCGCGCAAGGTGATTGCCGATCTGGGTTACGGCGATCAGCTCGGCGAAGATCCCGAC
GCGCAGCAAAACGACGAGGCCGATCAGGAGAGCGAGGAAGACCCGGAAGCGGATCAGGACAACCGCTCGGAGGCAGAGCAG
CAGGACGACGACGGCGAGGCCAGCCCCGAGCAAAGTCAGGAACAGCAGCAGGATCAGCGGCAAACCACCGTCAGCCAGGAC
GATCTCGCCGAGGACGAGAGCGGCGACGAGCTCGACATGCCGCAGGCCGATGCGCCGCCCGAACCGCCGCCGCCCGCGCCG
CATTCGAATGCCGATCCGAATTACACCGTCTTCACCACCGACTTCGACGAGGAAATCCGCGCCGAGGATCTGGCGGAACCG
GCGGAACTGGAACGGCTGCGCGCTTACCTTGACACGCAGCTCGAACCGCTGAAAGGCGCCGTCGCGCGGCTGGCGAACAAG
CTGCAACGGCGGCTGCAGGCGCAGCAGAACCGCAGCTGGGAATTCGACCGCGAGGAAGGCACGCTCGATGCTGGCCGCCTT
GCCCGCGTCGTGGCAAACCCGACGACGCCGCTGTCCTTCAAGATCGAGAAAGACACCGAATTCCGCGACACCTGCGTGACG
CTGCTTCTGGACAATTCGGGCTCGATGCGGGGCCGCCCGATTTCCATTGCCGCGATCTGCGCCGACGTGCTGGCGCGGACG
CTCGAACGCTGCTCGGTCAAGGTCGAGATCCTGGGCTTCACCACCCGCGCCTGGAAGGGCGGCCAAAGCCGCGAGAAATGG
CTGGCCGAGGGGCGCAAGCAGGGGCCGGGGCGGCTCAATGACCTGCGCCACATCATCTACAAATCCGCCGATGCGCCCTGG
CGGCGGGTGCGGTCCAATCTGGGCCTGATGATGAAGGAAGGCCTGCTGAAGGAAAACATCGACGGCGAGGCGCTGGAATGG
GCGTGGAAGCGGTCGATGGCGCGGCCCGAGGCGCGGAAAATCCTGATGGTGATTTCCGACGGCGCCCCGGTCGATGATTCG
ACGCTTTCGGTCAACCCCGCGATCTATCTGGAAAAACACCTGCGCGACGTGATCGCCATGGTGGAACGCCGCCGCGCGGTC
GAACTTCTGGCGATCGGCATCGGCCATGACGTGACGCGCTATTATTCGCGCGCCGTCACCATCACCGATGTCGAGCAGCTG
GCGGGCGCGATGACCGAACAGCTGGCGGCGCTTTTCGACAGCGATCCGCGGGCGCGGGCGCGGGTGCTGGGGATGAAAAAG
ACGGGGTAAGCGGGCATGTTTCAGGATTTCACCAGCCGCTCGACGCCTGACTAGTTTTGGATCC

Figure S10: DNA sequence of pET3a-CobST vector between BglII and SpeI restriction sites (including coding regions for CobS and CobT highlighted grey).

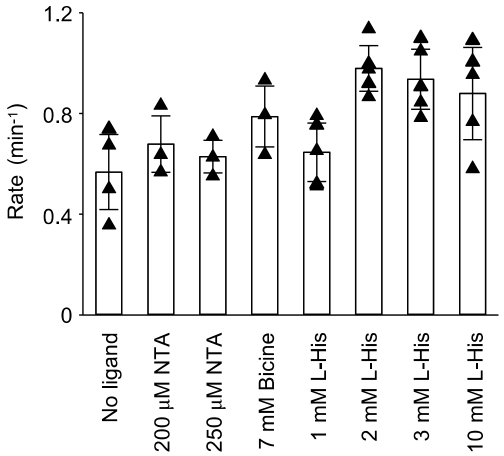

Figure S11: Rates of metalation of SHC by CbiK with different Co^{II}-buffering ligands. Measured rate of Co^{II} insertion into SHC by enzyme CbiK when identity of Co^{II} buffering ligand was varied: Note that where ligand = 200 μM and 250 μM NTA, data also appear in Figure 3 and are repeated here to facilitate easy rate comparison between ligands. Reactions performed in 50 mM Hepes buffer pH 7.0, 100 mM NaCl with [SHC] = 5 μM, [CbiK] = 0.375 μM, $[Co^{II}]tot$ = 100 μM and $[ligand]_{tot}$ as labelled. Data are the mean ± s.d. of n ≥ 3 replicates (triangles represent individual experiments). The available $[Co^{II}]$ in each reaction was >10 nM (ie >100-fold greater than Co^{II}-binding affinity of CbiK-SHC) thus the enzyme complex can be considered Co^{II}-saturated in each case. Slightly elevated reaction rates were observed when $[His]_{tot}$ > 1 mM, whereas increased buffering would have been predicted to reduce activity. It is formally possible that, when present at high concentrations, His may participate in a complex involving the enzyme and alter the reaction kinetics. Intracellular [His] in a bacterial cytosol (*Synechocystis* PCC 6803) were previously determined to be ~ 45 μM[9], thus [His] > 1mM are not physiologically relevant in this context. Therefore, [His] > 1 mM were not used for studies of CbiK.

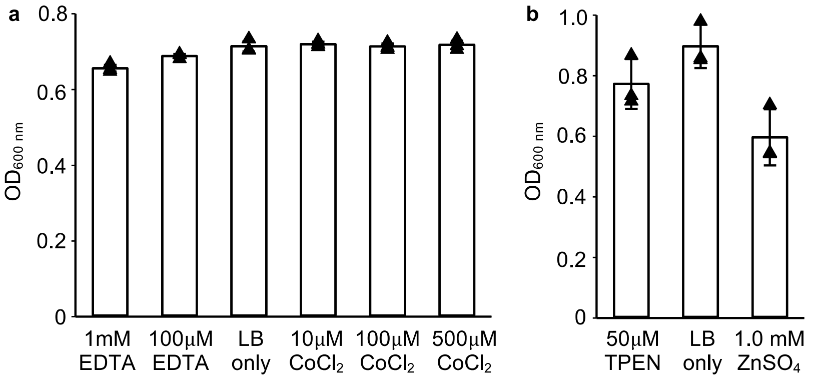

Figure S12: Optical density of *Salmonella* cultures.
a Final $OD_{600\ nm}$ of *Salmonella* cultures following 3 h treatment with $CoCl_2$ or EDTA (added at $OD_{600\ nm}$ ~ 0.5) under anaerobic conditions. **b** Final $OD_{600\ nm}$ of *Salmonella* cultures following 1 h treatment with $ZnSO_4$ or TPEN (added at $OD_{600\ nm}$ ~ 0.3) under aerobic conditions. All data are the mean ± s.d. of n=3 biological replicates (triangles show individual experiments).

Calculation S1: Calculation of Co^{II} occupancies of CobW and CobNST in metal-transfer complex

The reaction describing a competition between CobW and CobNST for binding Co^{II} is:

$$\mathrm{Co^{II}CobW} + \mathrm{CobNST} \rightleftharpoons \mathrm{CobW} + \mathrm{Co^{II}CobNST} \quad (1)$$

The equilibrium constant for the metal exchange reaction is:

$$K_{\mathrm{ex}} = \frac{[\mathrm{Co^{II}CobNST}][\mathrm{CobW}]}{[\mathrm{Co^{II}CobW}][\mathrm{CobNST}]} = \frac{K_{\mathrm{D}}(CobW)}{K_{\mathrm{D}}(CobNST)} \quad (2)$$

Here we assume a model where 1:1 complexes between $Co^{II}Mg^{II}$GTP-CobW and CobNST-HBAD form and, upon formation, GTP hydrolysis occurs resulting in 1:1 complexes of $Co^{II}Mg^{II}$GDP-CobW:CobNST-HBAD. Considering these complexes only, the following constraints and mass balances apply:

$[P]_{tot}$ = $[CobW]_{tot}$ = $[CobNST]_{tot}$ (3)

$[Co^{II}]_{tot}$ = $[P]_{tot}$ (4)

$[CobW]_{tot}$ = [CobW] + $[Co^{II}CobW]$ (5)

$[CobNST]_{tot}$ = [CobNST] + $[Co^{II}CobNST]$ (6)

$[Co]_{tot}$ = $[Co^{II}CobW]$ + $[Co^{II}CobNST]$ (7)

Substituting (3) into (5) gives:

[CobW] = $[P]_{tot}$ - $[Co^{II}CobW]$

Combined with (4) and (7) gives:

$[P]_{tot}$ = $[Co^{II}]_{tot}$ = $[Co^{II}CobW]$ + $[Co^{II}CobNST]$

$\Rightarrow$[CobW] = $[Co^{II}CobW]$ + $[Co^{II}CobNST]$ - $[Co^{II}CobW]$

$\Rightarrow$ [CobW] = $[Co^{II}CobNST]$

And similarly

[CobNST] = $[Co^{II}CobW]$

Substituting into equation (2):

$$\frac{[\mathrm{Co^{II}CobNST}][\mathrm{CobW}]}{[\mathrm{Co^{II}CobW}][\mathrm{CobNST}]} = \left(\frac{[\mathrm{Co^{II}CobNST}]}{[\mathrm{Co^{II}CobW}]}\right)^2 = \frac{K_{\mathrm{D}}(\mathrm{CobW})}{K_{\mathrm{m}}(\mathrm{CobNST})}$$

where K_m is used as a proxy for K_D for the CobNST complex.

Thus, the distribution of Co^{II} between the binding site of CobW and the binding site of CobNST is:

$$\frac{[\mathrm{Co^{II}CobNST}]}{[\mathrm{Co^{II}CobW}]} = \sqrt{\frac{K_{\mathrm{D}}(CobW)}{K_{\mathrm{m}}(CobNST)}} = \sqrt{\frac{100\ \mathrm{nM}}{50\ \mathrm{nM}}} = \sqrt{2} = 1.414$$

This equates to 59% of Co^{II} transferred to CobNST-HBAD.

Calculation S2: Calculations to assess the influence ATP on available [Co^{II}] during CobNST enzymatic assays.

Reported metal dissociation constants for 1:1 complexes with ATP[10, 11]:

$K_{\mathrm{Mg(II)}} = 87\ \mu\mathrm{M}$

$$K_{\mathrm{Co(II)}} = 23\ \mu\mathrm{M}$$

Total metal and nucleotide concentrations used in enzyme assays:

$[\mathrm{Co^{II}}]_{\mathrm{tot}} = 100\ \mu\mathrm{M}$

$[\mathrm{Mg^{II}}]_{\mathrm{tot}} = 10\ \mathrm{mM}$

$[\mathrm{ATP}]_{\mathrm{tot}} = 5\ \mathrm{mM}$

Under the given reaction conditions, Co^{II} and Mg^{II} compete for binding to ATP as described by the following equation:

$$\mathrm{Co^{II}} + \mathrm{Mg^{II}ATP} \rightleftharpoons \mathrm{Mg^{II}} + \mathrm{Co^{II}ATP} \qquad (1)$$

The equilibrium constant for the metal exchange reaction is:

$$K_{\mathrm{ex}} = \frac{[\mathrm{Mg^{II}}][\mathrm{Co^{II}ATP}]}{[\mathrm{Co^{II}}][\mathrm{Mg^{II}ATP}]} = \frac{K_{\mathrm{Mg(II)}}}{K_{\mathrm{Co(II)}}} = 3.78 \qquad (2)$$

Given that Mg^{II} is present in large excess and has sufficiently high affinity to bind ATP stoichiometrically under the experimental conditions, the following approximations can be made:

$$[\mathrm{Mg^{II}}] \sim [\mathrm{Mg^{II}ATP}] \sim 5\ \mathrm{mM}$$

Substituting into equation (2) gives:

$$\frac{[\mathrm{Co^{II}ATP}]}{[\mathrm{Co^{II}}]} \sim 3.78$$

Which in combination with the mass balance:

$$[\mathrm{Co^{II}ATP}] + [\mathrm{Co^{II}}] = 100\ \mu\mathrm{M}$$

can be used to calculate available cobalt concentration:

$$3.78 \times [\mathrm{Co^{II}}] = 100\ \mu\mathrm{M} - [\mathrm{Co^{II}}]$$

$$[\mathrm{Co^{II}}] = \frac{100\ \mu\mathrm{M}}{4.78} = 20.9\ \mu\mathrm{M}$$

These calculations show that ATP has the capacity to buffer Co^{II} availability, but the nucleotide does not compete with the CobNST chelatase for Co^{II} binding under these experimental conditions (CobNST has K_m of 50 nM for Co^{II}). Enzyme assays in Figure 2 employed additional (stronger) Co^{II}

binding ligands (His and EGTA) to achieve free $[Co^{II}]$ << $1\ \mu M$, which enabled competition with CobNST.

There is some evidence of formation of 1:2 $Co^{II}ATP_2$ complexes at low $[metal]_{tot}$:$[ATP]_{tot}$ ratios[12] but to our knowledge such complexes have not been characterised for Mg^{II} and β values are not available. 1:1 stoichiometries for $Mg^{II}ATP$ complexes have been reported in metal titrations with similar (millimolar) concentrations of reagents as used in these assays[13]. Here, given the large excess of total metal (Mg^{II} plus Co^{II}) over ATP which limits the unligated [ATP] in solution, it is anticipated that the equilibria favour 1:1 metal complex formation.

Supplementary References

(1) Xiao, Z.; Wedd, A. G. The challenges of determining metal–protein affinities. *Nat. Prod. Rep.* **2010**, *27* (5), 768-789.
(2) Martell, A.; Smith, R. NIST Critically Selected Stability Constants of Metal Complexes Database 46, Version 8.0. U.S. Dept. of Commerce, NIST Standard Reference Data Program, Gaithersburg, MD. **2004**.
(3) Young, T. R.; Martini, M. A.; Foster, A. W.; Glasfeld, A.; Osman, D.; Morton, R. J.; Deery, E.; Warren, M. J.; Robinson, N. J. Calculating metalation in cells reveals CobW acquires Co^{II} for vitamin B_{12} biosynthesis while related proteins prefer Zn^{II}. *Nat. Commun.* **2021**, *12* (1), 1195.
(4) Foster, A. W.; Clough, S. E.; Aki, Z.; Young, T. R.; Clarke, A. R.; Robinson, N. J. Metalation calculators for *E. coli* strain JM109 (DE3): Aerobic, anaerobic and hydrogen peroxide exposed cells cultured in LB media. *Metallomics* **2022**, mfac058.
(5) Clough, S. E.; Osman, D.; Young, T. R.; Robinson, N. J. Hyperaerated metalation calculator for *E. coli* strain JM109 (DE3) grown in LB media. *bioRxiv* **2022**, DOI: 10.1101/2022.1109.1102.506343.
(6) Osman, D.; Martini, M. A.; Foster, A. W.; Chen, J.; Scott, A. J. P.; Morton, R. J.; Steed, J. W.; Lurie-Luke, E.; Huggins, T. G.; Lawrence, A. D.; Deery, E.; Warren, M. J.; Chivers, P. T.; Robinson, N. J. Bacterial sensors define intracellular free energies for correct enzyme metalation. *Nat. Chem. Biol.* **2019**, *15* (3), 241-249.
(7) Osman, D.; Foster, A. W.; Chen, J.; Svedaite, K.; Steed, J. W.; Lurie-Luke, E.; Huggins, T. G.; Robinson, N. J. Fine control of metal concentrations is necessary for cells to discern zinc from cobalt. *Nat. Commun.* **2017**, *8* (1), 1884.
(8) Raux, E.; Lanois, A.; Levillayer, F.; Warren, M. J.; Brody, E.; Rambach, A.; Thermes, C. *Salmonella typhimurium* cobalamin (vitamin B_{12}) biosynthetic genes: functional studies in *S. typhimurium* and *Escherichia coli*. *J. Bacteriol.* **1996**, *178* (3), 753-767.
(9) Foster, A. W.; Pernil, R.; Patterson, C. J.; Scott, A. J. P.; Pålsson, L.-O.; Pal, R.; Cummins, I.; Chivers, P. T.; Pohl, E.; Robinson, N. J. A tight tunable range for Ni(II) sensing and buffering in cells. *Nat. Chem. Biol.* **2017**, *13* (4), 409-414.
(10) Smith, R. M.; Martell, A. E. *Critical stability constants: Volume 2: Amines*; Platinum Press, New York, 1975.
(11) Walaas, E. Stability constants of metal complexes with mononucleotides. *Acta chem. scand* **1958**, *12* (528), 3.
(12) Granot, J.; Fiat, D. Proton magnetic resonance study of divalent metal ions binding to adenosine 5'-triphosphate. *J. Am. Chem. Soc.* **1977**, *99* (1), 70-79.
(13) Pecoraro, V. L.; Hermes, J. D.; Cleland, W. W. Stability constants of magnesium and cadmium complexes of adenine nucleotides and thionucleotides and rate constants for formation and dissociation of magnesium-ATP and magnesium-ADP. *Biochemistry* **1984**, *23* (22), 5262-5271.
